# *LITTIP*/*Lgr6*/HnRNPK complex regulates cementogenesis via Wnt signaling

**DOI:** 10.1038/s41368-023-00237-0

**Published:** 2023-08-09

**Authors:** Tiancheng Li, Han Wang, Yukun Jiang, Shuo Chen, Danyuan Huang, Zuping Wu, Xing Yin, Chenchen Zhou, Yuyu Li, Shujuan Zou

**Affiliations:** 1 State Key Laboratory of Oral Diseases & National Center for Stomatology & National Clinical Research Center for Oral Diseases & West China Hospital of Stomatology, Sichuan University, Chengdu, China; 2 Department of Orthodontics, Shanghai Ninth People’s Hospital, Shanghai Jiao Tong University School of Medicine; College of Stomatology, Shanghai Jiao Tong University; National Center for Stomatology; National Clinical Research Center for Oral Diseases; Shanghai Key Laboratory of Stomatology; Shanghai Research Institute of Stomatology, Shanghai, China

**Keywords:** RNA sequencing, RNA, High-throughput screening, Cell signalling, Differentiation

## Abstract

Orthodontically induced tooth root resorption (OIRR) is a serious complication during orthodontic treatment. Stimulating cementum repair is the fundamental approach for the treatment of OIRR. Parathyroid hormone (PTH) might be a potential therapeutic agent for OIRR, but its effects still lack direct evidence, and the underlying mechanisms remain unclear. This study aims to explore the potential involvement of long noncoding RNAs (lncRNAs) in mediating the anabolic effects of intermittent PTH and contributing to cementum repair, as identifying lncRNA-disease associations can provide valuable insights for disease diagnosis and treatment. Here, we showed that intermittent PTH regulates cell proliferation and mineralization in immortalized murine cementoblast OCCM-30 via the regulation of the Wnt pathway. In vivo, daily administration of PTH is sufficient to accelerate root regeneration by locally inhibiting Wnt/β-catenin signaling. Through RNA microarray analysis, lncRNA *LITTIP* (**L**GR6 **i**n**t**ergenic **t**ranscript under **i**ntermittent **P**TH) is identified as a key regulator of cementogenesis under intermittent PTH. Chromatin isolation by RNA purification (ChIRP) and RNA immunoprecipitation (RIP) assays revealed that *LITTIP* binds to mRNA of leucine-rich repeat-containing G-protein coupled receptor 6 (LGR6) and heterogeneous nuclear ribonucleoprotein K (HnRNPK) protein. Further co-transfection experiments confirmed that *LITTIP* plays a structural role in the formation of the *LITTIP*/*Lgr6*/HnRNPK complex. Moreover, *LITTIP* is able to promote the expression of LGR6 via the RNA-binding protein HnRNPK. Collectively, our results indicate that the intermittent PTH administration accelerates root regeneration via inhibiting Wnt pathway. The lncRNA *LITTIP* is identified to negatively regulate cementogenesis, which activates Wnt/β-catenin signaling via high expression of LGR6 promoted by HnRNPK.

## Introduction

Orthodontically induced root resorption (OIRR) is a serious complication during orthodontic treatment, which causes resorption of cementum and dentin.^1,2^ OIRR may lead to decreased tooth longevity, compromised clinical outcome, and even dysfunctional stomatognathic system, when timely therapeutic intervention was not adopted during clinical treatment. Cementum is a mineralized tissue that accumulates at the surface of tooth roots, serving as a crucial component of the periodontal attachment apparatus. The maintenance of cementum integrity is essential for the long-term health and functionality of teeth.^3,4^ During orthodontic practice, excessive orthodontic force, extended treatment duration, or patient susceptibility are tended to induce defective or destructed cementum, which causes further tooth dysfunction and external root resorption.^5^ Therefore, maintaining the integrity of cementum by regenerative approaches is a pivotal step in the management of OIRR.

Parathyroid hormone (PTH) is the major endogenous hormone that is responsible for calcium and phosphorus homeostasis in the body, and its effects on bone metabolism largely depends on the mode of administration.^6,7^ In the field of oral medicine, many researches have concentrated on the therapeutic potential of PTH on the repair and regeneration of periodontal tissues.^8–11^ A preliminary study has found that intermittent PTH increased cementum formation during odontogenesis of young Sprague-Dawley rats.^12^ Accordingly, in vitro studies verified that intermittent PTH promoted cementogenesis- and differentiation-related biomarkers of cementoblasts.^13,14^ Intermittent PTH has also been found to enhance periodontal healing with increased formation of cementum-like tissues, in periodontal defects caused by injury or during orthodontic tooth movement.^10,13,15^ Although these data indicate that intermittent PTH administration might be an effective approach for cementum repair, the mechanisms underlying its therapeutic effects remain unclear.

Long noncoding RNAs (lncRNAs) are RNA molecules that are greater than 200 nucleotides in length without the property of protein-coding. Although initially regarded as merely transcriptional “noise”, lncRNAs have been uncovered to participate in numerous biological processes, as well as many human diseases.^16^ As a novel intervention target, versatile lncRNAs-related gene regulation facilitates the development of molecular drugs for diseases.^17,18^ Recently, the role of the lncRNAs in periodontal injury and regeneration was widely reported.^19,20^ LncRNA GACAT2 was discovered as a critical regulator of mitochondrial function and cementogenesis in an inflammatory environment.^21^ Under compressive force, upregulated lincRNA-p21 was found to inhibit cementoblast mineralization by impeding autophagy.^22^ These data indicate that lncRNAs are participated in biological processes of cementoblasts and may be involved in cementogenesis. Hence, we hypothesized that the lncRNA-associated mechanisms are essential for cementum repair after OIRR, and certain lncRNAs might mediate the therapeutic effects of intermittent PTH.

To clarify whether and how intermittent PTH affects the biological processes of cementoblasts, we analyzed the cementogenic related markers, mineralization, cell proliferation and apoptosis, as well as the expression profiles of both lncRNAs and mRNAs in OCCM-30 cells under PTH administration. We used a root resorption and regeneration model to verify that intermittent PTH promotes root regeneration via Wnt/β-catenin signaling. Using lncRNAs microarrays and correlation analysis, lncRNA *LITTIP* (**L**GR6 **i**n**t**ergenic **t**ranscript under **i**ntermittent **P**TH) was identified as a key regulator of cementogenesis under intermittent PTH. ChIRP assays, RIP experiments and co-transfection experiments demonstrated the role of *LITTIP* in promoting the expression of leucine-rich repeat-containing G-protein coupled receptor 6 (LGR6) by binding heterogeneous nuclear ribonucleoprotein K (HnRNPK). Our study identified the *LITTIP*/*Lgr6*/HnRNPK complex as a potential target to regulate cementogenesis via Wnt signaling.

## Results

### Intermittent PTH promotes cementogenesis but inhibits proliferation of OCCM‐30 cells

**Fig. 1:**
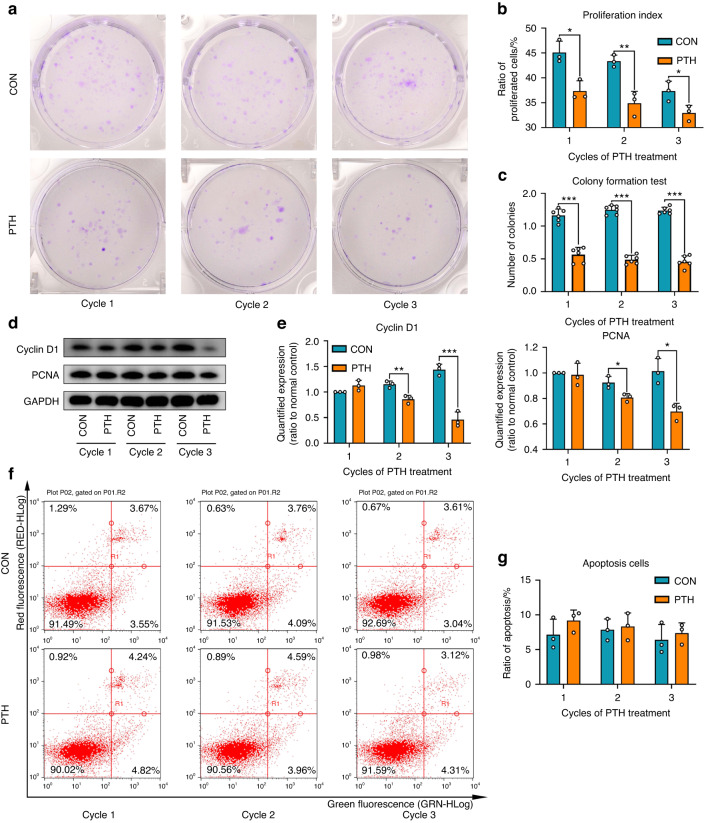
Intermittent PTH inhibits cell proliferation of OCCM-30 cells. **a** Crystal violet staining of OCCM-30 cells after 5 days of cultivation. **b** The proliferation index of PTH group was decreased by 1–3 cycles of intermittent PTH. *n* = 3; *P < 0.05, **P < 0.01. **c** The number of colony forming cells were decreased by administration of intermittent PTH. *n* = 6; ***P < 0.001. **d** Western blot analyses revealed that the expression of Cyclin D1 and PCNA were decreased by intermittent PTH. **e** Quantification was performed to show the protein changes of Cyclin D1 and PCNA. *n* = 3; *P < 0.05, **P < 0.01, ***P < 0.001. **f** After 1–3 cycles of intermittent PTH, cells were collected for apoptosis analysis using a flow cytometer. **g** Intermittent PTH had no effects on the ratio of apoptosis cells. *n* = 3

Based on the results of qRT-PCR and western blot, the expression of cementogenic related markers cementum attachment protein (CAP), Runx2, alkaline phosphatase (ALP), osteocalcin (OCN), and Osterix (Osx) was significantly increased by 1–3 cycles of PTH administration (Supplementary Fig. S1a–c). The results of Alizarin Red S (ARS) staining showed that the administration of intermittent PTH resulted in more mineralized nodules formation (Supplementary Fig. S1d, e). ALP staining indicated more ALP activities in PTH group (Supplementary Fig. S1f, g). These results revealed that intermittent PTH facilitated cementogenesis in vitro, which are in line with previous observations.^13^ Crystal violet staining revealed that the colony forming cells were significantly reduced by PTH treatment (Fig. 1a, c). Consistently, flow cytometry demonstrated that the proliferation index declined in PTH group, indicating greater number of cells in the G0/G1 phase (Fig. 1b and Supplementary Fig. S2a). The expression of proliferation-associated protein Cyclin D1 and PCNA was also diminished in the PTH group (Fig. 1d, e). However, there was no significant difference of the apoptosis ratio between groups after each cycle of PTH administration (Fig. 1f, g).

### Cementogenic related Wnt/β-catenin signaling was suppressed by intermittent PTH in OCCM‐30 cells

**Fig. 2:**
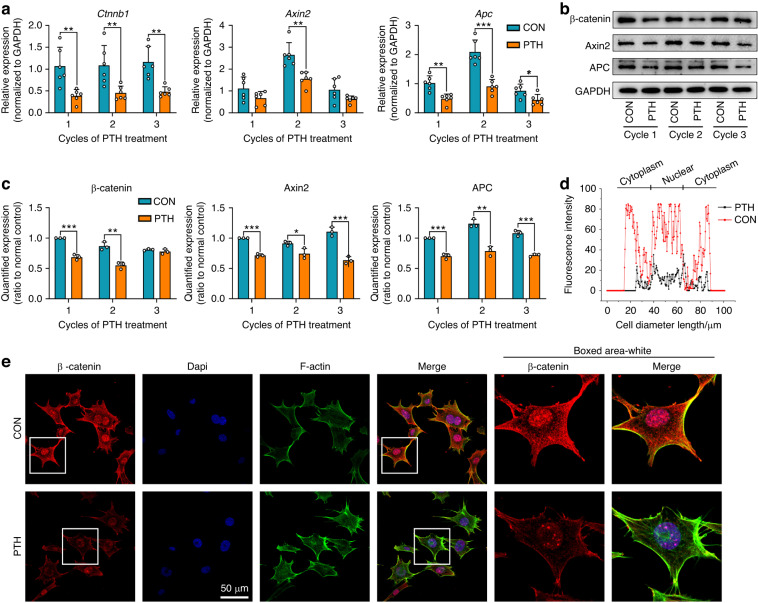
Intermittent PTH regulated the gene network of Wnt/β-catenin signaling in OCCM‐30 cells. **a** Relative mRNA expression of Wnt-related genes. The mRNA expression of β-catenin, Axin2 and APC were all decreased upon exposure to PTH treatment. *n* = 6; *P < 0.05, **P < 0.01, ***P < 0.001. **b** Western blot analyses revealed that the expression of β-catenin, Axin2 and APC was decreased with intermittent PTH application. **c** Quantification was performed to show the protein changes of β-catenin, Axin2 and APC. *n* = 3; *P < 0.05, **P < 0.01, ***P < 0.001. **d** Linear fluorescent quantification was performed to show the distribution of β-catenin cross the cell body in both groups. **e** Immunofluorescence staining of β-catenin. The β-catenin labeling was impaired at the presence of intermittent PTH. Scale bar: 50 µm

To analyze the impact of intermittent PTH on the gene expression in OCCM-30, an RNA microarray was performed (Supplementary Fig. S3a; GEO: GSE207523). Among the top 10 KEGG pathways enriched in sets of mRNAs, the Wnt signaling pathway (mmu04310) is among specific pathways which related to cementogenic and osteogenic differentiation (Supplementary Fig. S3b).^23,24^ A heatmap further revealed that gene expression negatively-related to canonical Wnt signaling, such as Draxin, Ctnnbip, Lrp1, Tle4, Nfatc4, Fzd1, Sox17 was upregulated, while gene expression positively-related to canonical Wnt signaling, such as Lgr6, Lef1, Wnt4, Fzd3 was downregulated in PTH group (Supplementary Fig. S3c). Through qRT-PCR and western blot assays, it was verified that the expression levels of Wnt signaling components such as β-catenin, Axin2 and APC were all significantly declined upon exposure to PTH treatment (Fig. 2a–c). Immunofluorescence showed that the β-catenin localized in both the cytoplasm and nucleus of no-PTH treated cells. β-catenin accumulation at the nuclear region was significantly decreased at the presence of intermittent PTH (Fig. 2d, e).

### Intermittent PTH promotes root regeneration via suppression of Wnt/β-catenin signaling

**Fig. 3:**
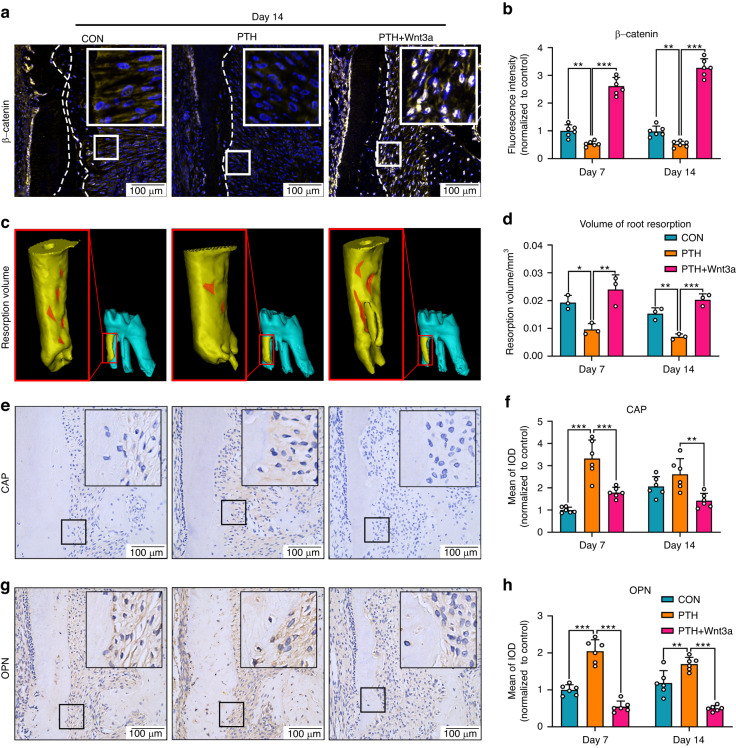
Intermittent PTH promotes root regeneration via regulation of Wnt/β-catenin signaling. **a**, **b** Immunofluorescence staining on the compression side of distal buccal root (**a**). Dashed lines show the outline of the root surface. Boxed area indicates region that is shown in detail. Scale bar: 100 μm. β-catenin+ (yellow) stained cementoblasts decreased with the application of intermittent PTH and accumulated after the application of Wnt3a (**b**). *n* = 6; *P < 0.05, **P < 0.01, ***P < 0.001. **c**, **d** Micro-CT analysis of distal buccal root of maxillary first molars (**c**). Boxed area indicates region that is shown in detail. Red shadows show the areas of root resorption. Quantitative analysis indicated that the volume of root resorption decreased by the intermittent PTH and increased after the application of Wnt3a (**d**). *n* = 3; *P < 0.05, **P < 0.01, ***P < 0.001. **e**, **g** Representative immunohistochemical images of cementogenesis‐related factors on the compression side of distobuccal roots. Positive staining of CAP and OPN was detected in the cementum and the presumed cementoblasts lining on it. Scale bar: 100 μm. Boxed area indicates region that is shown in detail. **f** Quantitative analysis indicated that the expression of CAP increased in PTH group during the first 7 days of repair, and declined by Wnt3a administration after 7 and 14 days of repair. *n* = 6; **P < 0.01, ***P < 0.001. **h** The OPN expression was increased by intermittent PTH, while decreased by Wnt3a administration on both day 7 and 14. *n* = 6; **P < 0.01, ***P < 0.001

We next examined whether the impact of PTH on canonical Wnt signaling contributed to the repair of root resorption and expression of cementogenic related markers after force removal. Immunofluorescence staining demonstrated that the expression level of β-catenin in cementoblasts was significantly diminished by intermittent PTH after 7 and 14 days of repair. As expected, increased β-catenin expression and nuclear accumulation of β-catenin were shown in cementoblasts of PTH + Wnt3a group (Fig. 3a, b and Supplementary Fig. S4a). Histological changes of periodontal tissues were examined using HE and Masson’s trichrome staining. Just after the removal of force, external root resorption in all groups was obvious on the compression side of root in all groups with periodontal fibers compressed irregularly (Supplementary Fig. S5b, c). With the progress of root resorption repair, regenerated periodontal fibers were aligned in a more orderly way, which gradually inserted into the newly formed cementum. After 14 days of root regeneration, intermittent PTH treatment essentially restored the root surface morphology, while small resorption lacunae were still presented on the CON and PTH + Wnt3a groups (Supplementary Fig. S5b, c). The dynamic changes of root resorption volumes were examined through micro-CT analysis. Although the volumes of root resorption showed a decreasing trend in all groups, the resorption volume of the PTH group was reduced faster than the other two groups both at day 7 and day 14, indicating highly efficient regeneration rate (Fig. 3c, d and Supplementary Fig. S4b). Finally, expression of cementogenesis‐related markers was analyzed using immunohistochemical staining. We found that the expression of CAP was increased in PTH group on day 7 and decreased by Wnt3a administration after 7 and 14 days of repair (Fig. 3e, f and Supplementary Fig. S4c). The expression of osteopontin (OPN) was also enhanced by daily administration of PTH, while only weak immunolabeling was found in the PTH + Wnt3a group on both day 7 and 14 (Fig. 3g, h and Supplementary Fig. S4d). The expression of both Runx2 and COL-1 followed a similar trend among all groups during the repair process (Supplementary Fig. S6a–d). These results indicated that the intermittent PTH mediated root regeneration was depended on local suppression of Wnt/β-catenin signaling.

### Identification of Wnt/β-catenin signaling-related lncRNAs under intermittent PTH

**Fig. 4:**
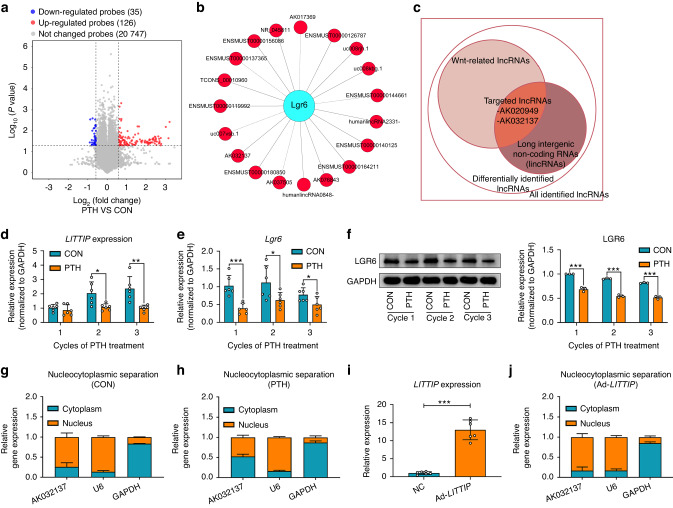
Identification of Wnt-related lncRNA *LITTIP* under intermittent PTH. **a** Volcano plot displays global lncRNA expression in the control and intermittent PTH sets. Blue represents downregulated lncRNAs; red represents upregulated lncRNAs. **b** Construction of lncRNAs-mRNAs correlation network. Wnt signaling related mRNA LGR6 was represented as blue circular nodes and lncRNAs were represented as yellow circular nodes. **c** Targeted lncRNAs were identified by taking intersection of the Wnt-related lncRNAs and the lincRNAs. Two lincRNAs (lncRNA AK032137 and AK020949) were included. AK032137 was further investigated in this study and named *LITTIP* (**L**GR6 **i**n**t**ergenic **t**ranscript under **i**ntermittent **P**TH). **d** Relative expression of *LITTIP* were decreased after 2–3 cycles of PTH treatment. *n* = 6; *P < 0.05, **P < 0.01. **e** Relative mRNA expression of LGR6 were decreased after 1–3 cycles of PTH treatment. *n* = 6; *P < 0.05, ***P < 0.001. **f** Western blotting and quantification showing the expression of LGR6 after 1–3 cycles of PTH treatment. *n* = 3; ***P < 0.001. **g**, **h** Cellular fractionation analyses showed that *LITTIP* was mainly located in the nucleus (**g**), and intermittent PTH decreased the expression of nuclear *LITTIP* (**h**). **i** Adenoviruses were used to overexpress *LITTIP* in OCCM-30 cells. Relative expression of *LITTIP* were remarkably increased. *n* = 6; ***P < 0.001. **j** Cellular fractionation analyses showed that adenoviruses increased the expression of nuclear *LITTIP*

Given that lncRNAs are key regulators in cementogenic activities,^21,22^ differentially expressed lncRNAs under intermittent PTH were identified (Fig. 4a). It is well known that long intergenic non-coding RNAs (lincRNAs) could directly regulate gene expression in cis,^25^ thus statistical analysis of differentially expressed lincRNAs and their nearby gene-derived mRNAs (<300 kb) was conducted. The results were shown in Supplementary Table S1. Additionally, a coding-noncoding gene co-expression (CNC) network was constructed to explore potential interactions between differentially expressed lncRNAs and Wnt signaling related mRNAs (Fig. 4b and Supplementary Fig. S7a). Taking the intersection of the above two analysis results, 2 lincRNA-mRNA pairs were included (Fig. 4c). In this study, we focused on AK032137 and named it *LITTIP* (**L**GR6 **i**n**t**ergenic **t**ranscript under **i**ntermittent **P**TH).

The results of qRT-PCR and western blot verification verified that consistent with findings from the microarray analysis, lncRNA *LITTIP* and its target gene *Lgr6* was both decreased after intermittent PTH (Fig. 4d–f). The subcellular localization of lncRNA *LITTIP* in OCCM-30 cells was further determined through nucleocytoplasmic separation of RNA. The results indicated that *LITTIP* distributed mainly in the nucleus, and intermittent PTH decreased the expression of nuclear *LITTIP* (Fig. 4g, h). From this, we speculated that lncRNA LITTIP did not exert its biological function via the competitive endogenous RNA mechanism, since it usually takes place in the cytoplasm. Given that the expression of *LITTIP* was significantly reduced by intermittent PTH, adenoviruses were used to overexpress its expression in OCCM-30 cells (Fig. 4i). We verified that the overexpressed *LITTIP* was principally localized in the nucleus (Fig. 4j).

**Fig. 5:**
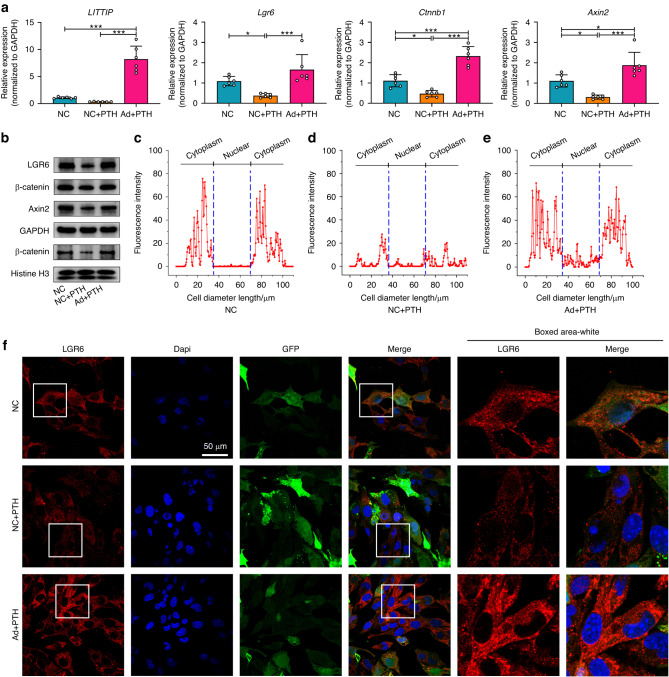
Intermittent PTH regulates Wnt/β-catenin signaling in OCCM‐30 cells via lncRNA *LITTIP*. **a** Relative mRNA expression of Wnt-related genes. The mRNA expression of LGR6, β-catenin, and Axin2 in OCCM-30 cells was decreased in the PTH group and upregulated by overexpression of *LITTIP*. *n* = 6; *P < 0.05, ***P < 0.001. **b** Western blot analyses revealed that the expression of β-catenin, Axin2, APC and nucleus β-catenin was decreased with intermittent PTH application and enhanced by adenovirus of *LITTIP*. **c**–**e** Linear fluorescent quantification was performed to show the intracellular distribution of LGR6 in OCCM-30 cells. **f** Immunofluorescence staining of LGR6. The LGR6 labeling was impaired at the presence of intermittent PTH and increased by overexpressed *LITTIP*. Scale bar: 50 µm

The overexpression of *LITTIP* reversed the inhibitory effects of intermittent PTH on LGR6 and enhanced the expression of β-catenin, Axin2, as well as nuclear β-catenin (Fig. 5a, b and Supplementary Fig. S8a). Consistently, immunofluorescence showed that the LGR6 and β-catenin labeling was increased by overexpression of *LITTIP*, and β-catenin accumulation at the nuclear region was significantly reduced at the presence of intermittent PTH (Fig. 5c–f and Supplementary Fig. S8b, c). To identify whether lncRNA *LITTIP* participated in cementoblastic mineralization, we examined the expressive changes of cementoblastic differentiation-related markers in OCCM-30 cells after the overexpression of *LITTIP*. We found that the expression of Runx2, Osx and CAP increased by intermittent PTH was significantly inhibited by overexpressed *LITTIP* (Supplementary Fig. S9a–c). In addition, ALP and ARS staining indicated significantly attenuated ALP activities and mineralized nodules formation under intermittent PTH in *LITTIP* overexpressed cementoblasts (Supplementary Fig. S9d, e). Furthermore, adenoviruses were injected on the periodontium near the maxillary left first molar for local *LITTIP* overexpression. We found that cementogenesis-related mRNAs Runx2, Osx and CAP in cementoblasts was upregulated by intermittent PTH and decreased significantly by in vivo overexpression of *LITTIP* (Supplementary Fig. S9f). These results indicated that the cementogenic effects of intermittent PTH were related to its inhibition of the lncRNA *LITTIP*.

### *LITTIP* directly binds to mRNA of *Lgr6* and HnRNPK protein

**Fig. 6:**
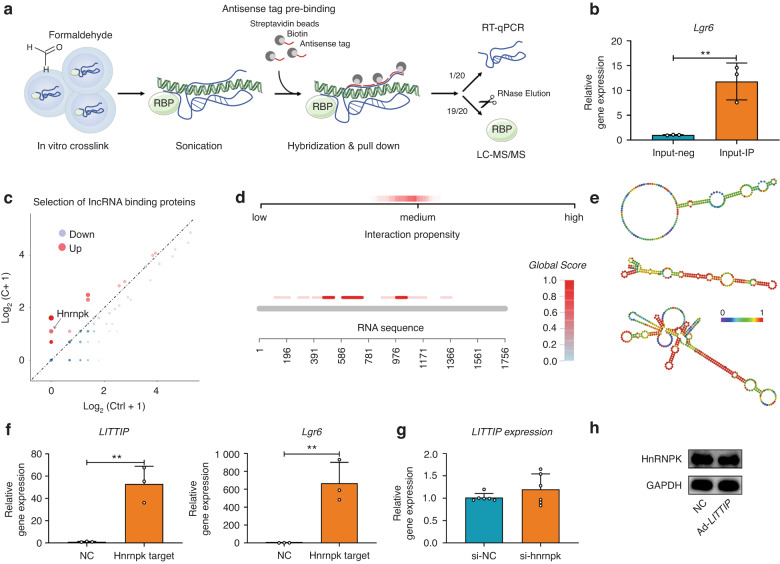
*LITTIP* directly binds to mRNA of *Lgr6* and HnRNPK protein. **a** The ChIRP method was applied to screen the potential RNAs and proteins binding to *LITTIP*. **b** The mRNA of *Lgr6* was enriched in the *LITTIP*-binding RNAs. *n* = 3; **P < 0.01. **c** Scatter plot displayed proteins precipitated by LITTIP probes. Red represents proteins with high binding specificity; blue represents proteins with low binding specificity. **d** Global Score analysis revealed a medium overall interaction ability between HnRNPK and lncRNA *LITTIP*. Three loci of *LITTIP* (461–532, 666–737, and 981–1052 nt) were predicted to interact with higher propensity. **e** Prediction of 461–532, 666–737, and 981–1052 nt *LITTIP* structure was based on minimum free energy (MFE) and partition function (http://rna.tbi.univie.ac.at/). **f** RIP experiment showed that lncRNA *LITTIP* and mRNA *Lgr6* were enriched in the HnRNPK-precipitated group compared with the IgG (control) group. *n* = 3; **P < 0.01. **g** Downregulation of HnRNPK failed to affect relative expression of *LITTIP*. *n* = 6. **h** Overexpression of *LITTIP* failed to affect protein level of HnRNPK

Then, a ChIRP-MS assay was used to identify potential *LITTIP*-binding RNAs and proteins (Fig. 6a). The mRNA of *Lgr6* was revealed to be enriched in the *LITTIP*-binding RNAs (Fig. 6b). Moreover, multiple complementary pairing sites were presented between two nucleic acids (Supplementary Fig. S10a), which further verified the direct binding between *LITTIP* and *Lgr6*. Among the 14 proteins enriched in the ChIRP lysate (Fig. 6c and Supplementary Table S2), HnRNPK was selected for further investigation due to the relation of HnRNPK with transcriptional regulation (based on the UniProt Database). Global Score revealed that the HnRNPK-*LITTIP* pair has medium overall interaction ability.^26^ *LITTIP* transcript at the 461–532, 666–737, and 981–1052 nt loci is predicted to interact with higher propensity (Fig. 6d). We further predicted the stem-loop structures of these loci, which is the necessary structural basis for the interaction with targeted RNA-binding proteins (Fig. 6e). Finally, the interaction of *LITTIP* with HnRNPK and *Lgr6* was verified by RIP experiment. We found that *LITTIP* and *Lgr6* was remarkably enriched in the HnRNPK-precipitated group compared to the IgG (control) group (Fig. 6f). Altogether, our results suggested that lncRNA *LITTIP* binds to and promotes the formation of *LITTIP*/*Lgr6*/HnRNPK complex.

### *LITTIP* promotes expression of LGR6 by binding *Lgr6* mRNA and HnRNPK protein

To investigate whether a reciprocal regulatory relationship existed among *LITTIP*, *Lgr6*, and HnRNPK, OCCM-30 cells were transfected with specific siRNA. The results revealed that si2-HnRNPK could most effectively knockdown HnRNPK (Supplementary Fig. S11a, b). Immunofluorescence staining revealed that the expression of HnRNPK at the nuclear region was significantly diminished (Supplementary Fig. S11c, d). Thus, si2-HnRNPK was chosen for further loss-of-function experiments. We found that HnRNPK downregulation fail to significantly affect *LITTIP* expression (Fig. 6g). Meanwhile, *LITTIP* overexpression fail to significantly affect HnRNPK expression (Fig. 6h and Supplementary Fig. S10b). Therefore, no significant interaction was found between *LITTIP* and HnRNPK.

**Fig. 7:**
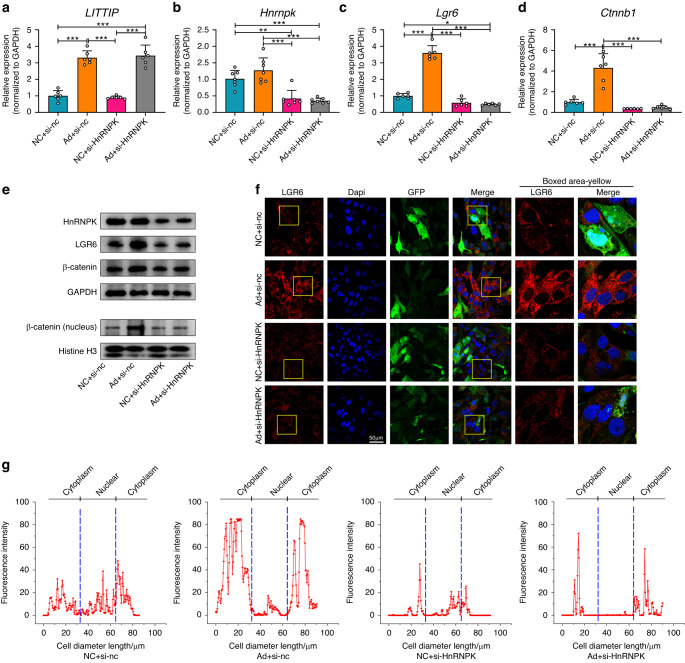
LncRNA *LITTIP* promotes expression of LGR6 via HnRNPK protein. **a**, **b** OCCM-30 cells were co-transfected with adenovirus of *LITTIP* and si-HnRNPK. Relative expression of *LITTIP* were remarkably increased in Ad + si-NC and Ad + si-HnRNPK groups (**a**). Relative mRNA expression of HnRNPK was decreased in NC + si-HnRNPK and Ad + si-HnRNPK groups (**b**). *n* = 6; **P < 0.01, ***P < 0.001. **c**, **d** Relative mRNA expression of LGR6 and β-catenin. The mRNA expression of LGR6 (**c**) and β-catenin (**d**) was upregulated by overexpression of *LITTIP* and decreased by si-HnRNPK. Downregulation of HnRNPK reversed the effects of *LITTIP* overexpression on mRNA expression of LGR6 and β-catenin. *n* = 6; *P < 0.05, ***P < 0.001. **e** Western blotting was performed to show relative protein expression of HnRNPK, LGR6, β-catenin and nucleus β-catenin. The expression of LGR6, β-catenin and nucleus β-catenin was enhanced by adenovirus of *LITTIP*. Downregulation of HnRNPK reversed the increased expression of LGR6, β-catenin and nucleus β-catenin by *LITTIP* overexpression. **f** Immunofluorescence staining of LGR6. Scale bar: 50 µm. **g** Linear fluorescent quantification was performed to show the cellular distribution of LGR6 in OCCM-30 cells. The LGR6 labeling was enhanced by adenovirus of *LITTIP* and this effect was impaired when HnRNPK was downregulated by siRNA

We speculated that the *LITTIP* might act as a scaffold in the formation of *LITTIP*/*Lgr6*/HnRNPK complex, promoting the transcription and maturation of *Lgr6* mRNA via HnRNPK. To verify this assumption, OCCM-30 cells were co-transfected with adenovirus of *LITTIP* (Fig. 7a) and si-HnRNPK (Fig. 7b). We found that downregulation of HnRNPK reversed the up-regulation effect of *LITTIP* overexpression on mRNA and protein expression levels of LGR6 and β-catenin, and nuclear distribution of β-catenin (Fig. 7c-e and Supplementary Fig. S12a). Immunofluorescence staining of LGR6 and β-catenin showed a completely consistent trend (Fig. 7f, g and Supplementary Fig. S12b, c).

**Fig. 8:**
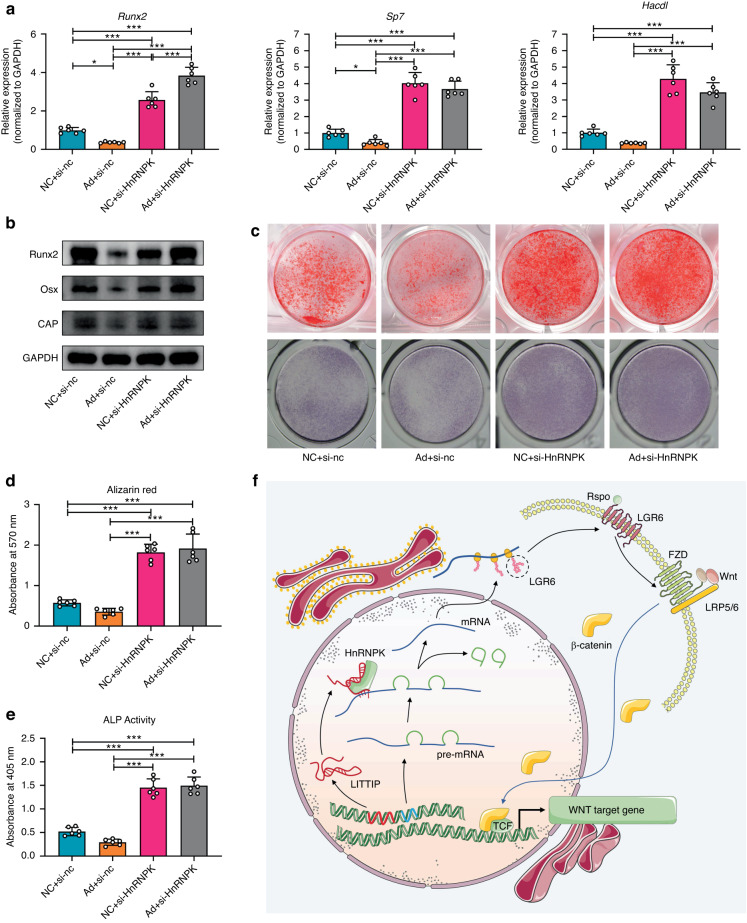
*LITTIP*/*Lgr6*/HnRNPK complex plays a crucial role in regulating cementogenesis. **a** Relative mRNA expression of cementogenesis-related genes. The mRNA expression of Runx2, Osx and CAP in OCCM-30 cells was decreased by overexpression of *LITTIP*, and increased when the complex function was inhibited by si-HnRNPK. *n* = 6; *P < 0.05, ***P < 0.001. **b** Western blotting showed the protein levels of Runx2, Osx and CAP in OCCM-30 cells. **c**–**e** Representative imag**e**s and quantitative analysis of ALP and ARS staining. *n* = 6; ***P < 0.001. **f** Schematic diagram showed that *LITTIP* promoted LGR6 expression via the *LITTIP*-binding protein HnRNPK

To identify whether the regulation of *LITTIP*/*Lgr6*/HnRNPK complex was linked to cementoblastic mineralization, the expression of cementogenic-related markers was also determined. The mRNA and protein levels of Runx2, Osx and CAP were inhibited by activated *LITTIP*/*Lgr6*/HnRNPK complex in Ad+si-nc group, and increased significantly when the complex function was inhibited in both the NC+si-HnRNPK and Ad+si-HnRNPK groups (Fig. 8a, b and Supplementary Fig. S13a). Accordingly, the results of ALP and ARS staining showed similar trends (Fig. 8c–e). These results indicated that *LITTIP* promoted LGR6 expression via the *LITTIP*-binding protein HnRNPK (Fig. 8f). The *LITTIP*/*Lgr6*/HnRNPK complex has the potential in regulating cementogenesis under intermittent PTH.

## Discussion

Although promoted cementogenesis by intermittent PTH has been validated in mature murine cementoblasts OCCM-30,^12,14^ precise signal that mediates this mineralization effect remains elusive. In this study, the global effects of intermittent PTH on the gene expression profiles of OCCM-30 cells were analyzed by high-throughput microarray. Among the top enriched KEGG pathways, the Wnt signaling was a specific downregulated pathway closely related to cementoblastic growth and differentiation.^27^ The inhibitory effect of intermittent PTH on Wnt/β-catenin signaling was verified through examining the expression levels of Wnt signaling components. In addition, Wnt pathway could partly mediate the anabolic effects of intermittent PTH on bone formation.^28^ The present evidence indicates that the effects of intermittent PTH might be regulated primarily by Wnt/β-catenin signaling. Interestingly, our in vitro results indicated that intermittent PTH inhibited the proliferation of OCCM-30 cells, with diminished expression of Cyclin D1 and PCNA. We therefore speculated that intermittent PTH could regulate cell proliferation and mineralization via the inhibition of Wnt pathway. Previous studies have reported results consistent with our findings, demonstrating that Wnt signaling activation suppresses cementoblast mineralization and differentiation,^29^ whereas inhibition of the Wnt/β-catenin pathway facilitates cementum formation.^30,31^ Our hypothesis is that Wnt pathway activation mainly facilitates cementoblast cell proliferation, while simultaneously suppressing cell differentiation, leading to a reduction in cell mineralization. Our study results suggest that moderate Wnt signaling inhibition via intermittent PTH treatment is the most effective approach to promote cementogenesis in OCCM-30 cells. This is probably due to an optimal balance between cell proliferation and differentiation, which created appropriate conditions for cementoblast mineralization.

Previous studies reported that conditional β-catenin stabilization in murine dental mesenchyme contributed to the differentiation of odontoblasts and cementoblasts, excessive dentin and cementum formation, and aberrant dento-alveolar complex during root development.^32,33^ In Wnt/β-catenin loss‐of‐function models, disrupted formation of cementoblasts was evident with rootless molars as well as incomplete incisors.^34,35^ The above studies illustrated that although canonical Wnt signaling was essential to cementum formation, overactivation of Wnt disfavored cementum mineralization and formation of ordered periodontal structures. To test whether a similar regulatory mechanism of canonical Wnt signaling exists in the repair of root resorption, a root repair model was used in the current study. Our in vivo results showed that the promoted root regeneration and cementogenic factors by intermittent PTH depended on the inhibited β-catenin level. Moreover, local upregulation of the Wnt/β-catenin pathway through Wnt3a could reverse the therapeutic effects of PTH. Here we showed that relative inhibition of canonical Wnt signaling was also beneficial for physiological cementum repair.

LncRNAs are known to participate in multiple biological processes, including periodontal tissue injury and regeneration.^19–22^ Our study investigated the role of lncRNAs in cementogenesis under intermittent PTH treatment. In particular, differentially expressed lincRNAs, lncRNA not overlapping a protein-coding transcript, were first screened as the potential research object for their diverse regulatory functions.^25,36^ Among the two Wnt related lincRNAs, *LITTIP* was identified as a key lncRNA related to the intermittent PTH promoted cementogenesis, since further gain-of-function studies showed that *LITTIP* overexpression caused Wnt activation in OCCM-30 cells and in turn contributed to impaired cementoblastic differentiation. LGR6, target gene of lncRNA *LITTIP*, is a facultative Wnt receptor component that regulate Wnt signal enhancement through soluble R-spondin proteins.^37^ Acting as crucial regulators of gene network, various lncRNAs mediate osteogenic potential of periodontal ligament cells to induce regeneration of dental tissues during inflammation.^38,39^ However, the effect of lncRNAs related mechanisms on cementogenesis remains mysterious in OIRR. We demonstrated that the inhibitory effects of intermittent PTH on canonical Wnt signaling relied on decreased *LITTIP* and LGR6 expression. This suggested that *LITTIP* has potential as a therapeutic target for OIRR treatment.

In the present study, as lncRNA *LITTIP* principally localized in the nucleus of the OCCM-30 cells, we speculated that *LITTIP* might exert its main biological effects through interactions with related proteins. To determine how functional lncRNA *LITTIP* regulate cementogenesis, we first screened and identified all the potential proteins that may interact with lncRNA *LITTIP*. Through RIP experiment, we further confirmed that *LITTIP* could directly bind to mRNA of LGR6 and HnRNPK protein. As one of the major pre-mRNA-binding proteins, HnRNPK has been shown to play a vital role in various aspects of gene regulation, including transcriptional and post-transcriptional mechanisms.^40^ It has been reported that the HnRNPK participates in the regulation of multiple genes,^41,42^ and can interact with β-catenin to alter downstream gene activation of the Wnt pathway.^43^ Although no significant interaction was found between *LITTIP* and HnRNPK, our subsequent co-transfection experiment confirmed that *LITTIP* promoted the transcription and maturation of *Lgr6* mRNA via binding with HnRNPK. Based on our findings, it appears that *LITTIP* is crucial to the formation of a *LITTIP*/*Lgr6*/HnRNPK complex, indicating its involvement in various aspects of gene regulation.

Although our findings demonstrate the pharmacological effects of intermittent PTH on the treatment of root resorption and identify lncRNA *LITTIP* as a new therapeutic target for promoting cementum regeneration after OIRR, there are still some limitations that should be addressed in future studies. Firstly, the specific binding site of HnRNPK on LGR6 mRNA and its mechanisms of promoting LGR6 expression need to be further explored. Secondly, the functional validation of *LITTIP* in in vivo experiments is not sufficient. In our future research plans, transgenic mice models should be constructed to further validate the potential role of *LITTIP* in the treatment of root resorption, and novel treatment system based on targeted regulation of lncRNA could be established accordingly.

In summary, our research uncovers the role of intermittent PTH in regulating the biological activities of cementoblasts and the repair of OIRR. Administration of intermittent PTH is sufficient to accelerate root regeneration and regulates the balance between cell proliferation and mineralization via the inhibition of Wnt pathway. The lncRNA *LITTIP* is identified as a key regulator of cementogenesis under intermittent PTH. *LITTIP* plays a structural role in the formation of *LITTIP*/*Lgr6*/HnRNPK complex and promotes expression of LGR6 via HnRNPK. Our results could provide a foundation for developing new therapeutic strategies for regenerating dental tissues.

## Materials and methods

### Cell culture, drug administration, and cell transfection

The immortalized murine cementoblast cell line OCCM-30 was maintained in Dulbecco’s modified Eagle medium (DMEM) with 5% fetal bovine serum (FBS). The intermittent PTH administration on OCCM-30 cells was performed as previously described.^12^ After 1–3 cycles of PTH treatment, cell samples were collected for further analyses. For lncRNA *LITTIP* overexpression, OCCM-30 cells were transfected with adenoviruses constructed by Haixing Biosciences (Suzhou, Jiangsu, China). After transfection for 48 h, cells were harvested and tested. To knockdown the HnRNPK expression, OCCM-30 cells were transiently transfected with specific siRNA oligonucleotides using RNAFit (Hanbio, Shanghai, China). These sequences were listed in (Supplementary Table S3). After transfection for 48 h, cells were harvested and tested.

### Flow cytometry

Using a commercial cell cycle kit (KeyGen Biotech, Nanjing, China), cell numbers at each stage were quantified using a flow cytometer (Guava, Merck Millipore, Germany). Proliferation index, indicating the rate of cell proliferation and DNA synthesis, was calculated using the formula (S + G2/M)/(G0/G1 + S + G2/M) × 100%. For apoptosis analysis, cells were collected and analyzed by flow cytometry (Guava, Merck Millipore, Germany) using an Apoptosis Detection Kit (Dojindo, Kumamoto, Japan).

### Crystal violet staining

Cells were seeded into 6-well plates with or without administration of intermittent PTH. After 5 days of cultivation, cells were stained with crystal violet solution (0.1%) for 10 min.

### Immunofluorescence and confocal laser scanning microscopy (CLSM)

Cells were fixed, permeabilized with 0.5% Triton X-100 (Beyotime, Shanghai, China), and blocked with 5% BSA. Primary antibodies specific to β-catenin (dilution 1:200, EM0306, Huabio, Hangzhou, China), LGR6 (dilution 1:200, ab126747, Abcam, Shanghai, China) and HnRNPK (dilution 1:200, SC60-03, Huabio, Hangzhou, China) were then added and incubated overnight at 4 °C. Secondary antibodies labeled with Cy3 (A0521, Beyotime, Shanghai, China) or Alexa Fluor 555 (ab150074, Abcam, Shanghai, China) were used to incubate the samples for 2 h. DAPI (S2110, Solarbio, Beijing, China) and phalloidine (6 μmol/L, Invitrogen, Carlsbad, CA, USA) were applied to stain the nuclei and cytoskeleton. Finally, cell samples were observed with CLSM.

### Bioinformatics analysis

The Kyoto Encyclopedia of Genes and Genomes (KEGG) (http://www.genome.jp/kegg/) was used to analyze differentially expressed mRNAs in order to identify important signal pathways. A coding-noncoding gene co-expression (CNC) network was constructed to assess the interactions between differentially expressed mRNAs and lncRNAs. The lncRNAs and mRNAs that exhibited a correlation coefficient >0.9 were selected to construct the network using Cytoscape 3.6 (Institute of Systems Biology, Seattle, USA).

### Nucleocytoplasmic separation

Isolation of cytoplasmic cytonuclear RNA and protein from cells was performed using the PARIS^TM^ Kit (Invitrogen, Carlsbad, CA, USA). The RNAs extracted from nuclear and cytoplasmic fractions were purified and reverse transcribed, and qRT-PCR was then performed using standard protocols. The extracted RNAs were identified using nuclear markers (U6) and cytoplasmic markers (GAPDH). Moreover, the expression of lncRNAs along with nuclear (U6) and cytoplasmic references (GAPDH) in each fraction was normalized to their levels in the RNA samples from whole cells, which was set to 100%.^44^

### Chromatin isolation by RNA purification (ChIRP) assay

ChIRP assays were conducted using *LITTIP*-specific biotin probes as previously described (Magna ChIRP, Millipore, USA).^45^ The enzymatic hydrolysate was separated by nano-UPLC liquid phase system (EASY-nLC1200) and used for MS. The proteins precipitated by *LITTIP* probes were quantified based on the fold change of normalized spectral counts relative to the negative control. The ChIRP probe (*LITTIP*) sequences used are provided in Supplementary Table S4. The results of the ChIRP-MS assay are listed in Supplementary Table S5.

### RNA immunoprecipitation (RIP)

RIP experiments were carried out with the Imprint RIP Kit (Sigma-Aldrich, St. Louis, USA) using 5 μg of rabbit anti-HnRNPK antibody (ET1610-38, Huabio, Hangzhou, China) or rabbit IgG. The coprecipitated RNAs were detected by qRT‐PCR.

### Animals

The study used seventy-two 6-week-old male Wistar rats with a weight range of (200 ± 10) g. All experimental procedures were approved by the Ethics Committee of West China Hospital of Stomatology (WCHSIRB-D-2022-020) and were conducted in accordance with the ARRIVE guidelines for preclinical animal studies.

### Application of orthodontic devices and retention

The maxillary left first molar and incisor were connected using an orthodontic elastic closed-coil spring after the rats were anesthetized with pentobarbital sodium, and a force of 100 g was applied to induce OIRR, as previously described.^46,47^ Following a 2-week period, the orthodontic coil spring was taken out, and an orthodontic wire was subsequently positioned between the maxillary left first molar and the incisor for the purpose of retaining the tooth’s position. The beginning of the retention phase was defined as day 0.

### Injection of PTH or Wnt signaling activator

Since the beginning of retention phase, seventy-two rats were randomly assigned to one of three groups: the control group (CON), the PTH group (PTH), and the PTH + Wnt3a group (PTH + Wnt3a). The PTH group received a daily subcutaneous injection of PTH (1–34) (Bachem, Torrance, CA, USA) at a dosage of 4 μg per 100 g of body weight. The PTH + Wnt3a group was injected with same volume of PTH and received daily periodontal local injection of 10 μL Wnt signaling activator Wnt3a (PreproTech, Rocky Hill, New Jersey, USA) with a concentration of 200 ng·mL^−1^ on both the buccal and lingual side of periodontium near the maxillary left first molar. The CON group received only the vehicle. To evaluate the degree of root regeneration after orthodontic force application, six rats of each group were sacrificed after 0, 3, 7 and 14 days of retention. The alveolar bone block that included the left first molar was harvested for further analysis, and Supplementary Fig. S5a illustrates all the experimental procedures.

### In vivo transduction of cementoblasts by adenoviruses

At the beginning of retention phase, experimental rats were randomly divided into three groups of 6 animals each: the negative control group (NC), the PTH group (NC + PTH), and the PTH + adenoviruses group (Ad + PTH). The Ad + PTH group received a daily subcutaneous injection of PTH together with periodontal local injection of 10 μL adenoviruses constructed by Haixing Biosciences (Suzhou, Jiangsu, China) with a concentration of 1 × 10^10^ IFU/mL for local lncRNA *LITTIP* overexpression in vivo. The NC and PTH group received periodontal local injection of same concentration of negative control adenoviruses. Three rats of each group were sacrificed after 7 and 14 days of retention (*n* = 3). For further qRT‐PCR analysis, the layer of cells (presume cementoblasts) lining on the compression side of roots were collected and total RNA was extracted using a commercial extraction kit (Bioer Technology, Hangzhou, China) based on manufacturer’s protocol.

### Statistical analysis

This study used the SPSS 22.0 software package (IBM Corporation, Armonk, NY, USA) for statistical analysis. The data were expressed as mean ± standard deviation from at least three independent experiments. The statistical significance was evaluated using Student’s *t*-test and one-way analysis of variance (ANOVA). The threshold for statistical significance was set at *P* < 0.05.

### Supplementary information

Supplementary Information

## Data Availability

The data supporting the results of this study are available upon request from the corresponding authors. The lncRNA microarray data are available in the GEO databases (accession number GSE207523).
