# Supplementary material for: LITTIP/Lgr6/HnRNPK complex regulates cementogenesis via Wnt signaling
Source: Int J Oral Sci. 2023 Aug 9;15:33. doi: 10.1038/s41368-023-00237-0 (PMC10412570; doi:10.1038/s41368-023-00237-0)
Supplement: Supplementary file 1 — Supplementary Information [file 41368_2023_237_MOESM1_ESM.docx]

**Supplementary Information**

***LITTIP*/*Lgr6*/HnRNPK complex regulates cementogenesis via Wntsignaling**

Tiancheng Li^1,2^, Han Wang^1^, Yukun Jiang^1^, Shuo Chen^1^, Danyuan Huang^1^, Zuping Wu^1^, Xing Yin^1^, Chenchen Zhou^1^, Yuyu Li^1,*^, Shujuan Zou^1,*^

^1^State Key Laboratory of Oral Diseases & National Center for Stomatology & National Clinical Research Center for Oral Diseases & West China Hospital of Stomatology, Sichuan University, Chengdu, China.

^2^Department of Orthodontics, Shanghai Ninth People's Hospital, Shanghai Jiao Tong University School of Medicine; College of Stomatology, Shanghai Jiao Tong University; National Center for Stomatology; National Clinical Research Center for Oral Diseases; Shanghai Key Laboratory of Stomatology, Shanghai, China.

**Supplementary Information Includes the Following:**

**Supplementary Materials & Methods**

**SUPPLEMENTARY FIGURES (13)**

**SUPPLEMENTARY TABLES (6)**

**Supplementary Materials & Methods**

**Alizarin Red S staining and quantification**

For osteogenic differentiation, cells were cultured in mineralization medium containing ascorbic acid (50 μM), dexamethasone (100 nM), β-glycerophosphate (10 mM) (Sigma, St. Louis, MO, USA) and 5% FBS in DMEM. For intermittent PTH treatment, 100 ng/ml PTH was added to the mineralization medium for the first 3 cycles and then every 3 days. After 14 days of mineralization medium incubation, Alizarin Red S staining was performed to detect the mineralized nodules with Alizarin Red S Stain Solution (Cyagen,St. Clara, CA, USA) according to the manufacturer’s instructions. Mineralized nodules stained by Alizarin red were observed using an Olympus IX70 microscope (Olympus, Tokyo, Japan). For quantitative calcium measurement, 10% cetylpyridinium chloride (J&KCHEMICA, Beijing, China) solution was then added to each well for elution of the dye. After incubation and shaking at room temperature for 1 hour, samples of the resulting solution were separately loaded into a 96-well plate and read at 570 nm.

**ALP staining and quantitative assay**

The ALP activity was determined using an ALP Activity Assay (Beyotime, Shanghai, China) according to the manufacturer’s instructions.^1^ The results were visualized under a light microscope (Olympus IX71, Tokyo, Japan). For quantitative assay, cells were lysed with ultrasound. Supernatants were collected for ALP activity determination according to the quantitative ALP assay kit (Beyotime, Shanghai, China). The optical density (OD) was measured at 405 nm.

**Quantitative real‐time polymerase chain reaction (qRT‐PCR)**

The qRT‐PCR analysis was conducted with the Quant Studio 3 Real‐Time PCR Systems (Thermo Fisher Scientific,San Jose, CA, USA) to validate the expression of lncRNAs and mRNAs. Reverse transcription was performed by PrimeScript™ RT Reagent Kit with gDNA Eraser (Takara, Tokyo, Japan). qRT‐PCR reaction was performed using TB Green™ Premix Ex Taq™ II (Takara, Tokyo, Japan). Gene expression level was normalized against glyceraldehyde-3‐phosphate dehydrogenase (GAPDH). The mRNA and lncRNA primer sequences used in this study are listed in Supplementary Table S6. Relative gene expression was calculated using the 2^-△△Ct^ method.

**Western blot**

The OCCM-30 cells from each culture plate were scraped using sterile plastic cell scraper (AxeniaBiologix, Dixon, CA, USA). The total protein was extracted with lysis buffer (KeyGENBioTECH, Nanjing, China) and normalized using the BCA Protein Assay Kit (KGP902; KeyGENBioTECH, Nanjing, China). Membranes were blocked with 5% nonfat skim milk (BD Biosciences, San Jose, CA, USA) in TBST for 1 hour and incubated with primary antibodies at 4°C overnight. Primary antibodies included anti-cementum attachment protein (CAP, dilution 1:200, sc-53947) from Santa Cruz (Shanghai, China); anti-LGR6 (dilution 1:1000, ab126747) from Abcam (Shanghai, China); anti-Runx2 (dilution 1:200, ET1612-47), anti-COL-1 (dilution 1:200, ER63063), anti-osteocalcin (OCN, dilution 1:200, ER1919-20), anti-alkaline phosphatase (ALP, dilution 1:1000, ET1601-21), anti-Osx (dilution 1:1000, ER1914-47), anti-PCNA (dilution 1:1000, ET1605-38), anti-Cyclin D1 (dilution 1:1000, ET1601-31), anti‐β-catenin (dilution 1:1000, ET1601-5), anti‐Axin2 (dilution 1:1000, ET1703-96), anti‐APC (dilution 1:1000, ET1601-80), and anti-HnRNPK (dilution 1:1000, SC60-03) from Huabio (Hangzhou, China). Anti‐GAPDH (dilution 1:10000, EM1101) and anti‐Histone H3 (dilution 1:10000, A11-D7) from Huabio (Hangzhou, China) were used as internal controls. Proteins were detected using horseradish peroxidase‐conjugated secondary antibodies (ZSGB‐Bio, Beijing, China) at room temperature for 1 hour and visualized using Clarity^TM^ Western ECL Substrate (Bio‐Rad, Hercules, CA, USA). The intensity of each band was calculated after normalization to GAPDH or Histone H3.

**Microarray analysis**

The expression profiles of lncRNAs and mRNAs in OCCM-30 cells from the PTH group and control group were measured using the Arraystar Mouse LncRNA Microarray version 3.0. Three biological replicates were used for each group, and the cells were treated for 2 cycles as previously described.^44^ Differentially expressed lncRNAs and mRNAs were identified using volcano plot filtering and were considered significant if they had a fold change >1.5 and a P value <0.05. Hierarchical clustering was performed using Agilent Gene Spring GX software (version 11.5.1).

**Micro-computed tomography (micro-CT) analysis**

All the samples were collected and scanned using the high-resolution micro-CT 50 system (Scanco Medical, Brüttisellen, Switzerland) with a voxel resolution of 10 μm, passing through a 3-dimensional Gaussian filter (mean, 1.2; filter support, 1). Then, the Mimics 21.0 software was employed to reconstruct and split three-dimensional models of distal-buccal root of maxillary left first molar. The size of resorption lacunae on the mesial surface of the distal buccal root was calculated as the volume difference of the roots with and without the assumed surface as previously described.^2,3^

**Histological staining**

After micro-CT scanning, the left half of the maxilla of each animal was fixed and decalcified for paraffin embedding. Then, 5-μm serial sections were cut in a mesiodistal direction parallel to the long axis of the distal root of the first molar and mounted on glass slides. Selected sections were treated with hematoxylin and eosin (HE) (G1120, Solarbio, Beijing, China) and Masson's trichrome (G1340, Solarbio, Beijing, China) staining and examined under a light microscope (Nikon Eclipse 80i microscope, Toyko, Japan).

**Immunofluorescence staining**

Tissue sections were heated at 95°C for 30 min for antigen retrieval, washed with PBS for 5 min, and blocked in 4% bovine serum albumin for 30 min at 37°C. Then, the samples were incubated with primary antibodies of β-catenin (dilution 1:200, EM0306, Huabio, Hangzhou, China) overnight at 4°C. On the following day, slides were incubated with the Alexa Fluor 555 goat anti-mouse (ab150114, 1:200, Abcam, Shanghai, China) for 1 h at 37°C and counterstained with DAPI (S2110, Solarbio, Beijing, China) for 15 min. Stained sections were observed using a fluorescent microscopy (DMI 6000; Leica, Wetzlar, Germany). Quantitative analyses were done by the Image-Pro Plus 6.0 Software (Media Cybernetics, Bethesda, MD, United States).

**Immunohistochemistry**

Tissue sections were placed in 3% hydrogen peroxide for 30 minutes in the dark. Subsequently, sections were blocked in blocking solution containing 4% bovine serum albumin for 20 minutes to prevent nonspecific background staining. Then, sections were incubated with primary antibodies diluted in blocking solution at different optimized dilutions: CAP (dilution 1:50, sc-53947) from Santa Cruz (Shanghai, China); Runx2 (dilution 1:200, ET1612-47), COL-1 (dilution 1:200, ER63063) and osteopontin (OPN, dilution 1:200, 0806-6) from Huabio (Hangzhou, China), at 4℃ overnight and then 37 °C for one hour. After rinsing, the slides were incubated with goat anti-rabbit or goat anti-mouse IgG secondary antibody HRP conjugated (dilution 1:1000, L3013-2, L3032-2, Baltimore, College Park, USA) for 30min at 37 ̊C. The immune reaction was visualized by using a 3,3’-diaminobenzidine DAB kit (ZLI-9017, Zhongshan Bio-Tech, Beijing, China). Slides were counterstained with hematoxylin and viewed using a light microscope (Nikon Eclipse 80i microscope, Toyko, Japan). The regions of interest were defined asthe cementum in an apical third area of the compression side of the distal buccal root and the layer of cells (presume cementoblasts) lining on it.^4^ The means of integrated option density (IOD) of immunohistochemical staining were analyzed by Image‐Pro Plus 6.0 Software (Media Cybernetics, Bethesda, MD).

**Supplementary references**

1 Yu, L. *et al.* circ_0003204 regulates the osteogenic differentiation of human adipose-derived stem cells via miR-370-3p/HDAC4 axis. *Int J Oral Sci***14**, 30 (2022).

2 Li, T. *et al.* Effects of estrogen on root repair after orthodontically induced root resorption in ovariectomized rats. *Am. J. Orthod. Dentofacial Orthop.***158**, 247-263.e241 (2020).

3 Li, T. *et al.* Intermittent parathyroid hormone promotes cementogenesis via ephrinB2-EPHB4 forward signaling. *J. Cell. Physiol.***236**, 2070-2086 (2021).

4 Bosshardt, D. D. & Schroeder, H. E. Initial formation of cellular intrinsic fiber cementum in developing human teeth. A light- and electron-microscopic study. *Cell and tissue research***267**, 321-335 (1992).

**Supplementary Figures**


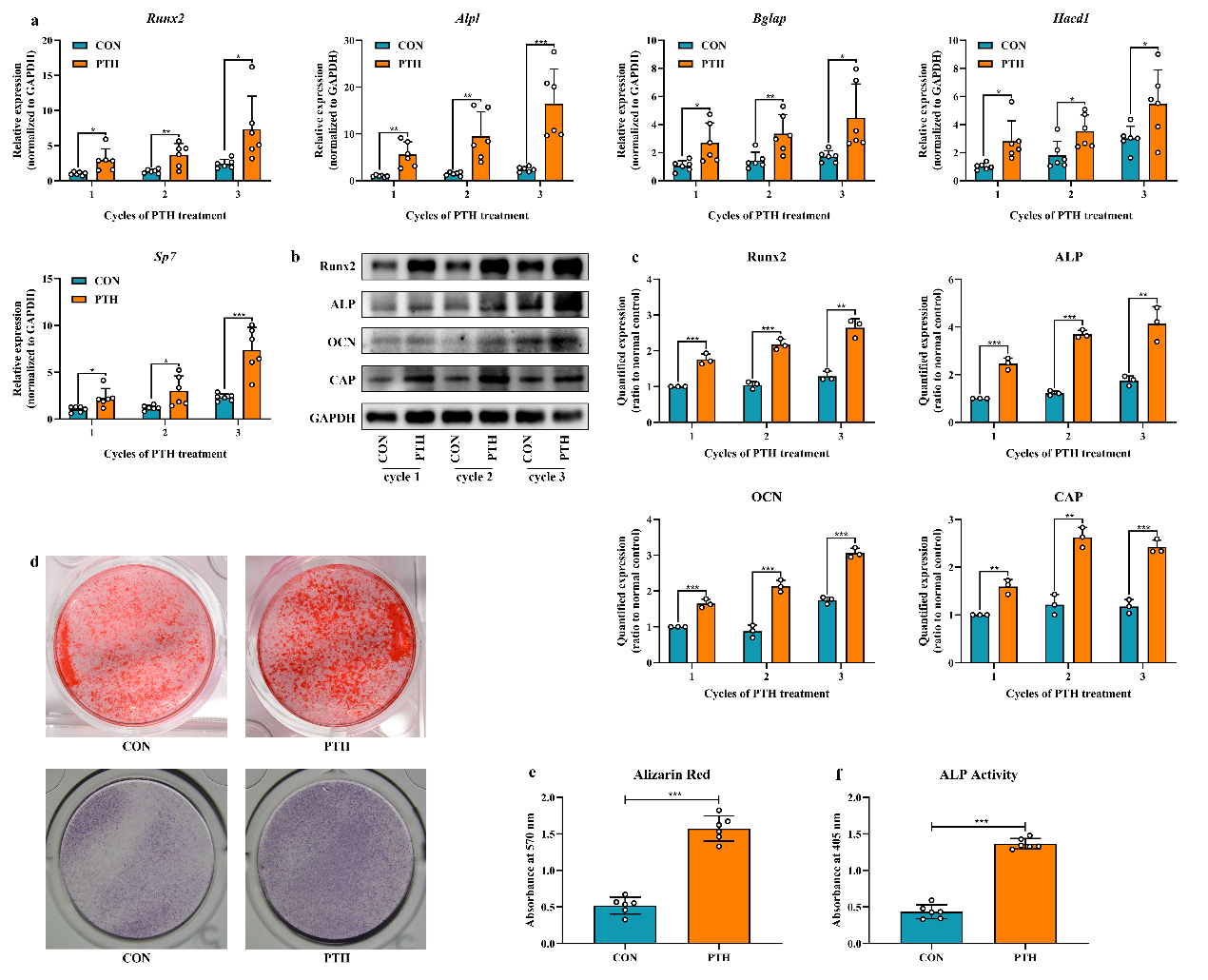


**Supplementary Fig. S1** Intermittent PTH promotescementogenesis of OCCM-30 cells. **a**Relative mRNA expression of cementogenic related markers. The mRNA expression of Runx2, ALP, OCN, CAP, and Osx in OCCM-30 cells was increased by 1-3 cycles of PTH treatment. n = 6; *P < 0.05, **P < 0.01, ***P < 0.001. **b** Western blot analyses revealed that the expression of Runx2, ALP, OCN and CAP was increased with intermittent PTH application. **c** Quantification was performed to show the protein changes of Runx2, ALP, OCN, and CAP. n = 3; **P < 0.01, ***P < 0.001. **d-f**ARS staining indicated more mineralized nodules formation in PTH group after 14 days of osteogenic induction. ALP staining revealed more active ALP activities in PTH group. n = 6; ***P < 0.001.


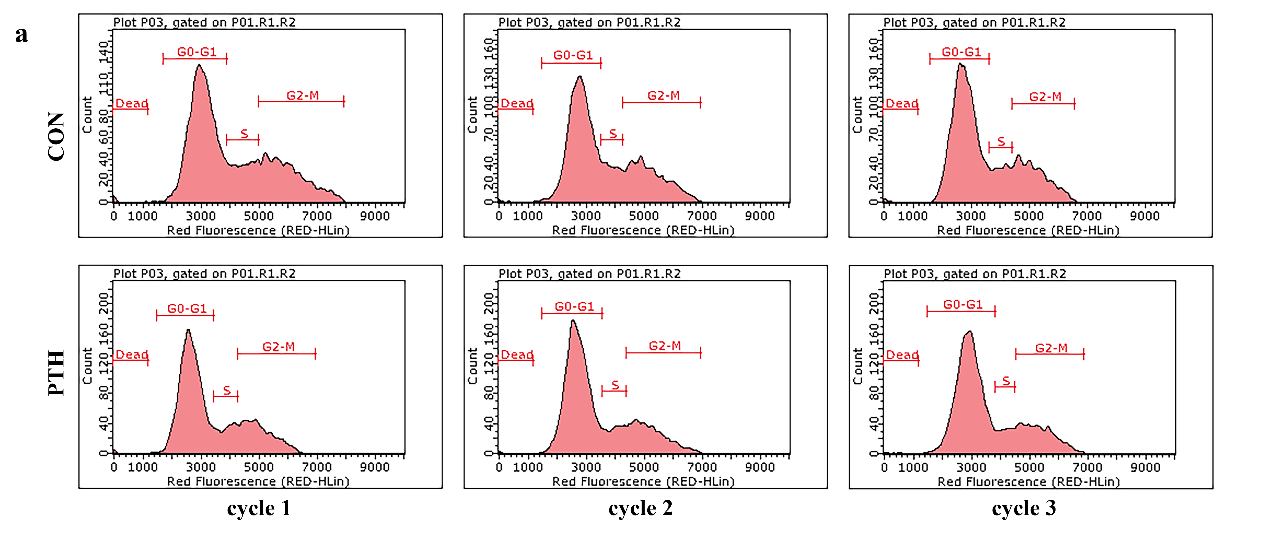
**Supplementary Fig. S2** Intermittent PTH inhibits cell proliferation of OCCM-30 cells. **a**After 1-3 cycles of intermittent PTH, cell numbers at each stage were determined using a flow cytometer.


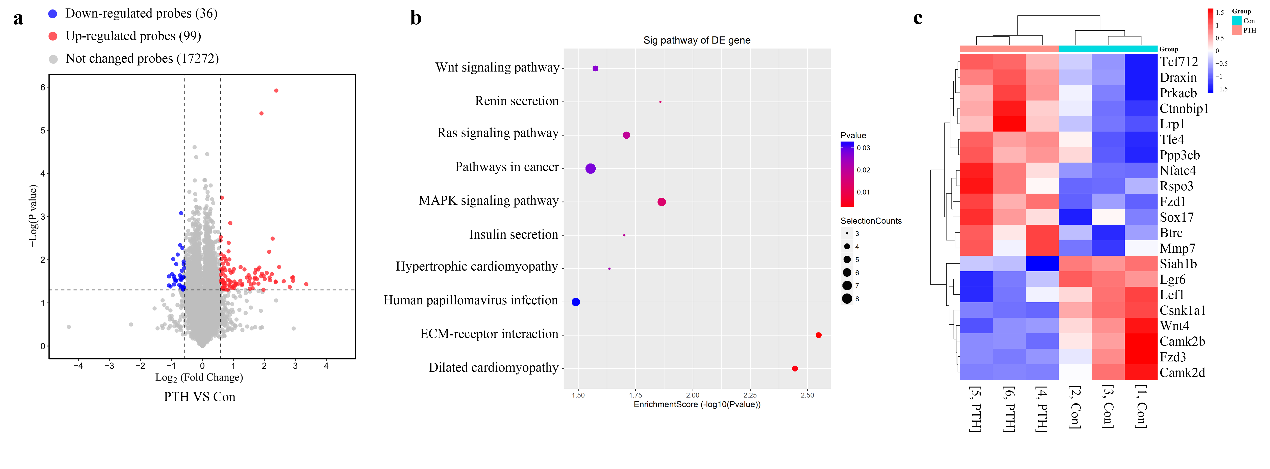


**Supplementary Fig. S3** Intermittent PTH regulated the gene network of Wnt/β-catenin signaling in OCCM‐30 cells. **a** Total RNA was isolated from OCCM-30 cells of control and intermittent PTH groups, followed by RNA microarray analysis. Volcano plot displays global gene expression in the control and intermittent PTH sets. Blue represents downregulated genes; red represents upregulated genes. **b** KEGG analysis of differentially expressed genes (>1.5-fold) between the two sets. **c** Heatmap analysis of Wnt-related genes in the control and intermittent PTH sets.


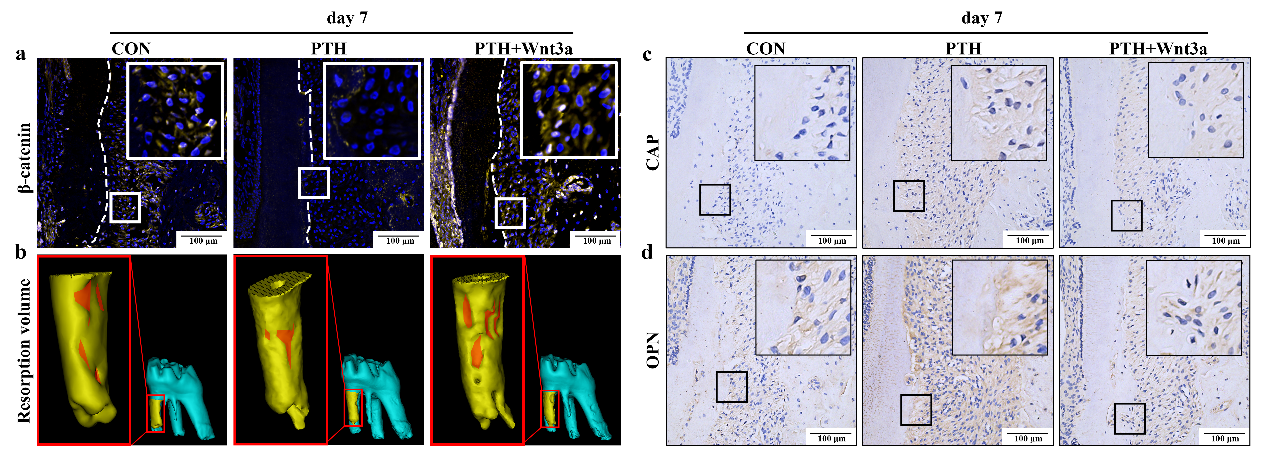


**Supplementary Fig. S4** Intermittent PTH promotes root regeneration via regulation of Wnt/β-catenin signaling.**a** Immunofluorescence staining on the compression side of distal buccal root on day 7. Dashed lines show the outline of the root surface. Boxed area indicates region that is shown in detail. Scale bar: 100 μm.**b** Micro-CT analysis of distal buccal root of maxillary first molars. Boxed area indicates region that is shown in detail. Red shadows show the areas of root resorption. **c, d** Representative immunohistochemical images of cementogenesis‐related factors on the compression side of distobuccal roots. Positive staining of CAP (c) and OPN (d) was detected in the cementum and the presumed cementoblasts lining on it. Scale bar: 100 μm. Boxed area indicates region that is shown in detail.


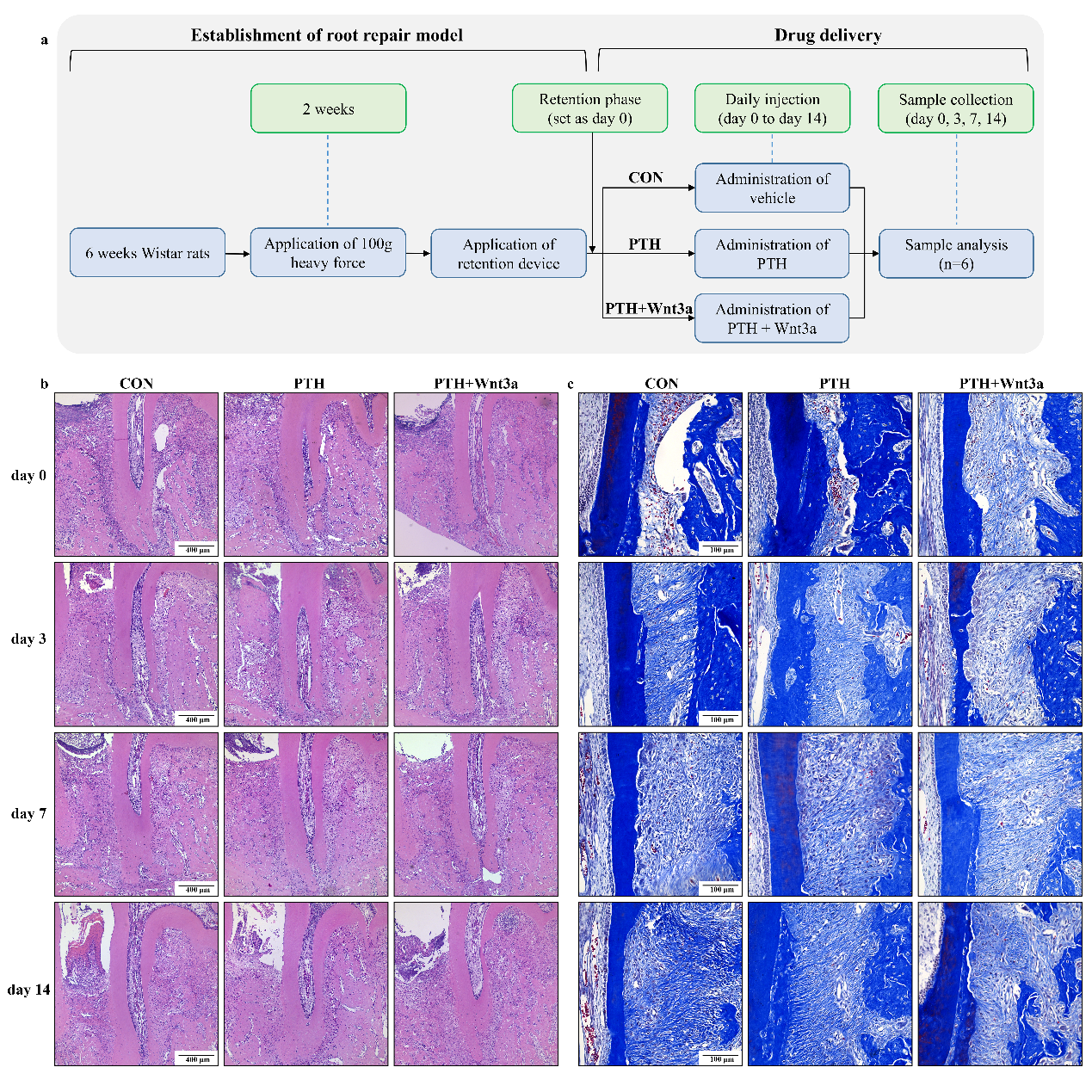


**Supplementary Fig. S5** Experimental design for animal study and histological change during root regeneration. **a** Flow chart of the experimental design for allocation of animals and schedule for drug administration. **b** HE staining of distal buccal roots of maxillary first molars showed that intermittent PTH treatment accelerated the regeneration of root surface morphology, while injection of Wnt3a delayed root regeneration. Scale bar: 400 µm. **c** Masson's trichrome staining revealed that periodontal fibers were compressed on day 0 and aligned more regularly along with the root repair process in all groups. Scale bar: 100 µm.


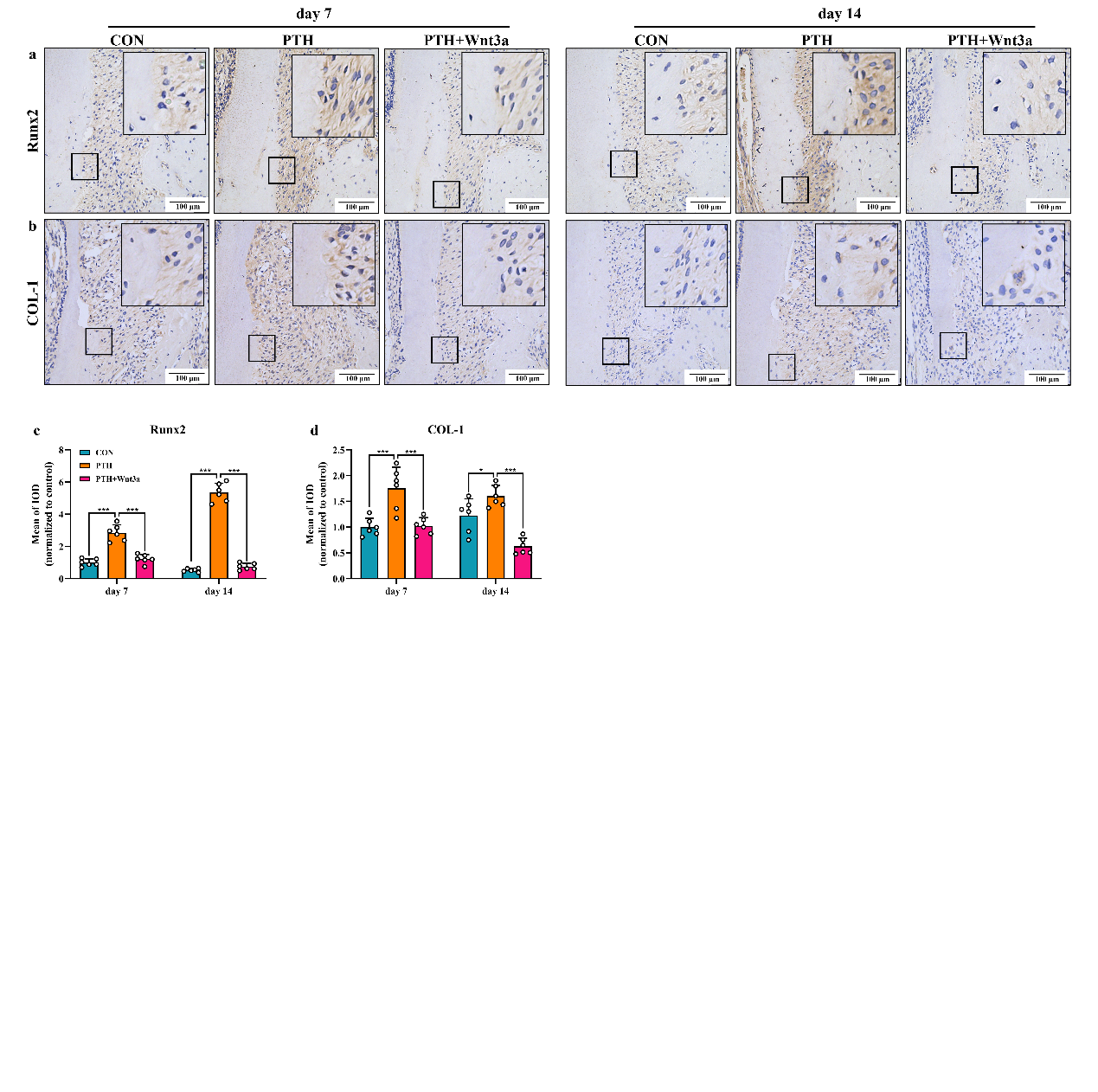


**Supplementary Fig. S6** Intermittent PTH promotes expression of Runx2 and COL-1 via regulation of Wnt/β-catenin signaling. **a, b** Representative immunohistochemical images of cementogenesis‐related factors on the compression side of distobuccal roots. Positive staining of Runx2 (a) and COL-1 (b) was detected in the cementum and the presumed cementoblasts lining on it. Scale bar: 100 μm. Boxed area indicates region that is shown in detail. **c** Quantitative analysis indicated that the expression of Runx2 was increased in PTH group and declined by Wnt3a administration after 7 and 14 days of repair. n = 6; ***P < 0.001. **d**The COL-1 expression was increased by intermittent PTH, while decreased by Wnt3a administration on both day 7 and 14. n = 6; *P < 0.05, ***P < 0.001.


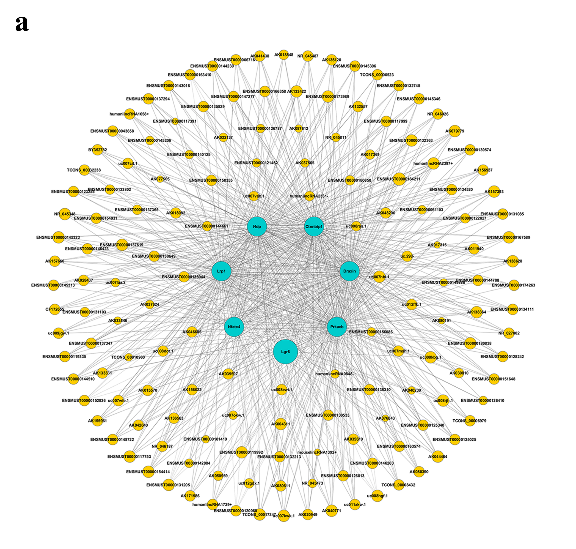


**Supplementary Fig. S7** Construction of lncRNAs-mRNAs correlation network. **a**Wnt signaling related mRNAs were represented as blue circular nodes and lncRNAs were represented as yellow circular nodes.


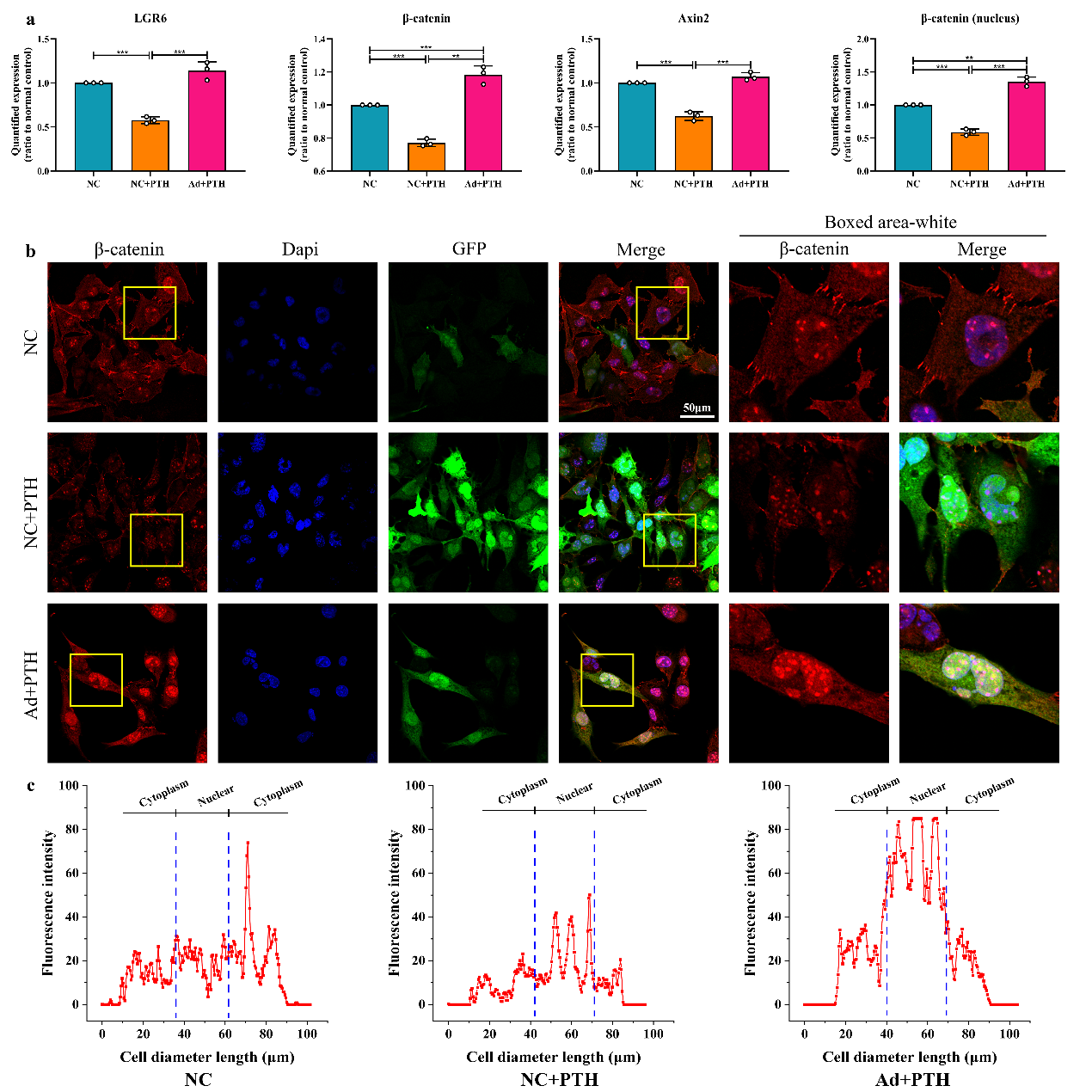


**Supplementary Fig. S8**Intermittent PTH regulates β-catenin in OCCM‐30 cells via lncRNA *LITTIP*.**a** Quantification was performed to show the protein changes of β-catenin, Axin2, APC and nucleus β-catenin. n = 3; **P < 0.01, ***P < 0.001. **b** Immunofluorescence staining of β-catenin. The expression of β-catenin was decreased by intermittent PTH and enhanced by adenovirus of *LITTIP*. Scale bar: 50 µm.**c** Linear fluorescent quantification was performed to show the cellular distribution of β-catenin.


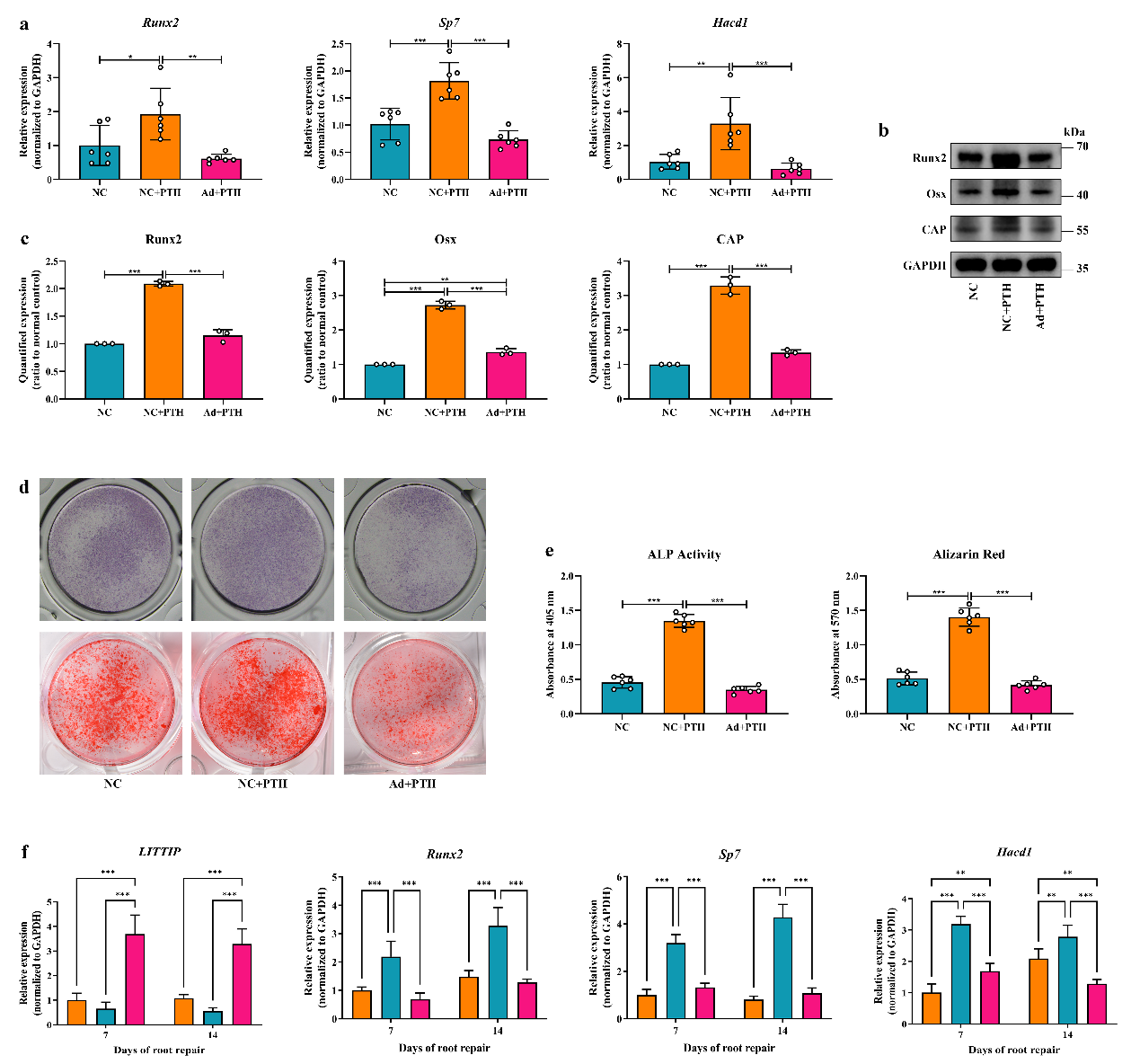


**Supplementary Fig. S9** Intermittent PTH promotes cementogenesis via regulation of *LITTIP*. **a** Relative mRNA expression of cementogenesis-related genes. The mRNA expression of Runx2, Osx and CAP in OCCM-30 cells was upregulated in the PTH group and decreased by overexpression of *LITTIP*. n = 6; *P < 0.05, **P < 0.01, ***P < 0.001. **b** Western blot analyses revealed that the expression of Runx2, Osx and CAP was increased with intermittent PTH and reduced by *LITTIP* overexpression. **c** Quantification was performed to show the protein changes of Runx2, Osx and CAP. n = 3; **P < 0.01, ***P < 0.001. **d** Representative images of ALP and ARS staining. **e** Quantitative analysis indicated that the ALP activities and mineralized nodules were promoted in PTH group and reduced by adenovirus of *LITTIP*. n = 6; ***P < 0.001.

**f**Relative expression of lncRNA *LITTIP* and cementogenesis-related mRNAs in cementoblasts after *in vivo* transduction by adenoviruses. The expression of Runx2, Osx and CAP was upregulated by intermittent PTH and decreased significantly by overexpression of *LITTIP*. n = 3; **P < 0.01, ***P < 0.001.


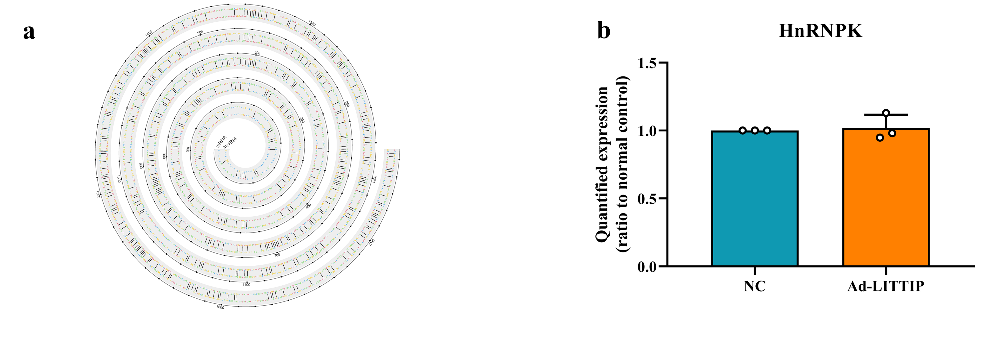
**Supplementary Fig. S10***LITTIP* directly binds to mRNA of *Lgr6* and HnRNPK protein. **a** Sequence analysis showed that multiple complementary pairing sites were presented between two nucleic acids. **b** Overexpression of *LITTIP* failed to affect protein level of HnRNPK.


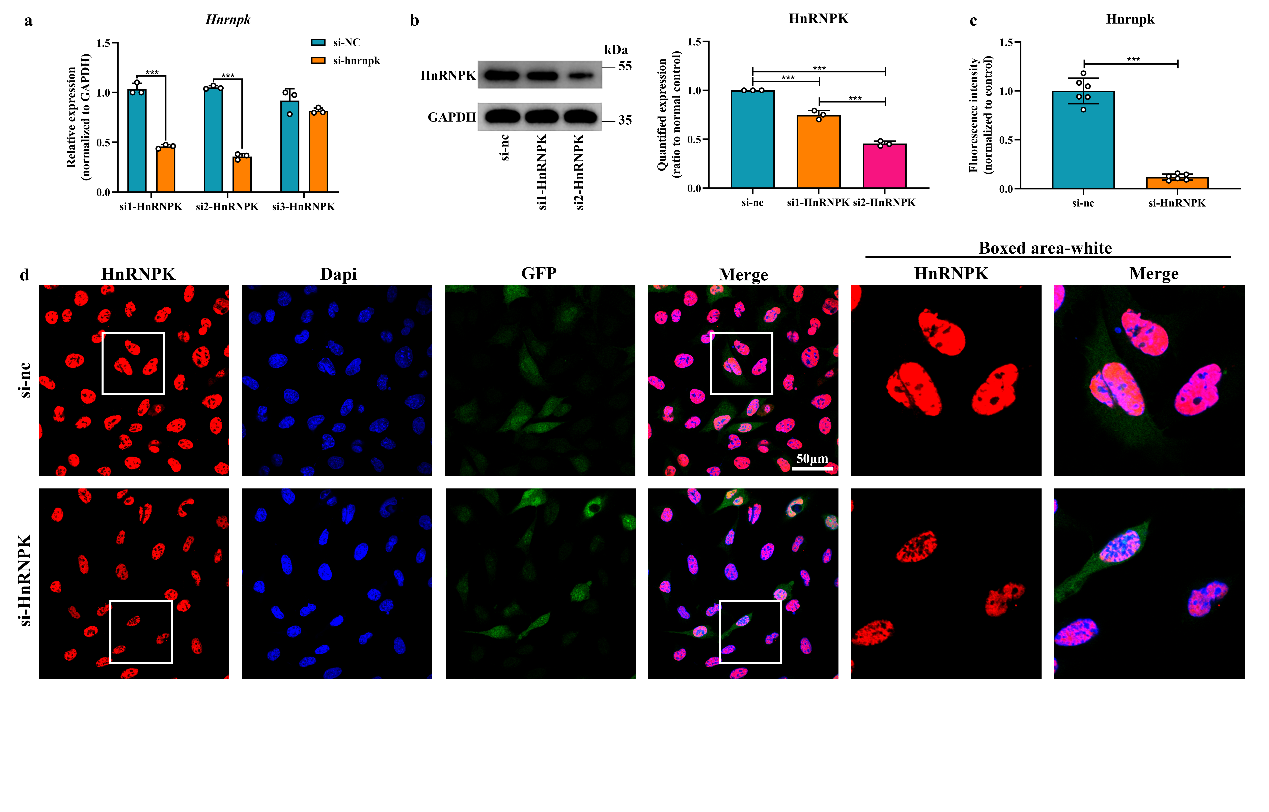


**Supplementary Fig. S11** Knockdown of HnRNPK in OCCM-30 cells. **a**The results of qRT-PCR revealed that relative mRNA expression of HnRNPK could be effectively reduced by si1-HnRNPK and si2-HnRNPK. n = 3; ***P < 0.001. **b** Western blotting and quantification revealed that si2-HnRNPK could most effectively knockdown protein level of HnRNPK. n = 3; ***P < 0.001. **c** Quantitative analysis indicated that the expression of HnRNPK was significantly declined by si-HnRNPK. n = 6; ***P <0.001. **d** Immunofluorescence staining of HnRNPK. The HnRNPK labeling was impaired at the presence of si-HnRNPK. Scale bar: 50 µm.


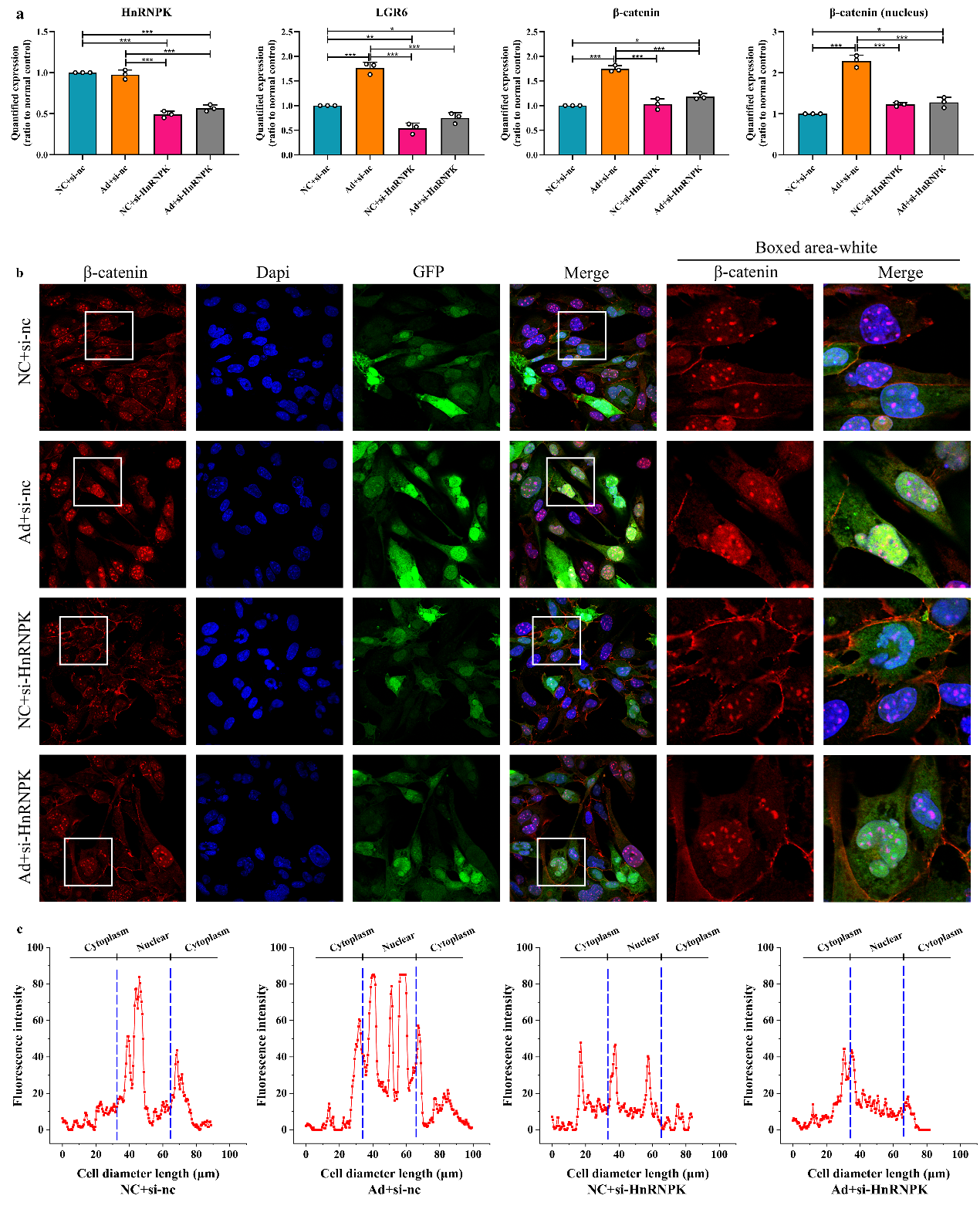
**Supplementary Fig. S12**LncRNA *LITTIP* promotes expression of β-catenin via HnRNPK protein. **a** Quantification was performed to show the protein changes of HnRNPK, LGR6, β-catenin, and nucleus β-catenin. n = 3; *P < 0.05, **P < 0.01, ***P < 0.001. **b** Immunofluorescence staining of β-catenin. The expression of β-catenin was upregulated by overexpression of *LITTIP* and decreased by si-HnRNPK. Downregulation of HnRNPK reversed the effects of *LITTIP* overexpression on immunolabeling of β-catenin. Scale bar: 50 µm.**c** Linear fluorescent quantification was performed to show the cellular distribution of β-catenin.


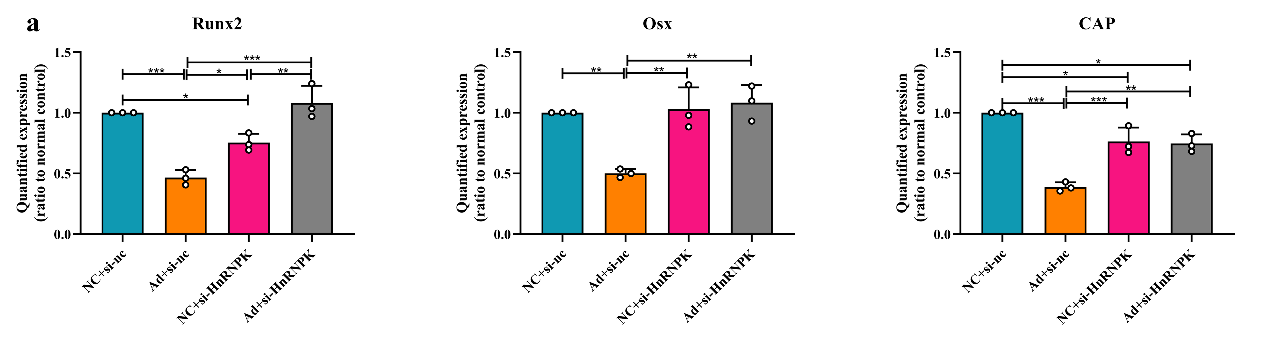


**Supplementary Fig. S13***LITTIP*/*Lgr6*/HnRNPK complex plays a crucial role in regulating cementogenesis. **a** Quantification was performed to show relative protein expression of Runx2, Osx, and CAP. n = 3; *P < 0.05, **P < 0.01, ***P < 0.001.

**Supplementary Tables**

**Supplementary Table S1** Characteristics of differentially expressed lincRNAs and their nearby mRNAs.

| Seqname | Gene Symbol | Regulation lncRNAs | Genome Relationship | Nearby Gene | Nearby Gene Symbol | Regulation mRNAs |
| --- | --- | --- | --- | --- | --- | --- |
| NR_045487 | 1700026F02Rik | up | downstream | NM_008353 | Il12rb1 | up |
| TCONS_00000523 | XLOC_001393 | up | upstream | NM_178598 | Tagln2 | down |
| AK020949 | AK020949 | up | upstream | NM_023465 | Ctnnbip1 | up |
| AK032137 | AK032137 | down | upstream | NM_001033409 | Lgr6 | down |
| uc008kbg.1 | BC024571 | down | upstream | NM_008397 | Itga6 | down |

**Supplementary Table S2**Proteins with high binding specificity.

| **Gene**  **names** | **Symbol** | **Number of proteins** | **Unique**  **peptides** | **Sequence**  **coverage** | **MS.MS**  **count** | **Q.value** | **Score** | **MS.MS**  **Count ctrl** | **MS.MS**  **Count Lnc** | **MS.MS**  **Count U1** | **iBAQ** |
| --- | --- | --- | --- | --- | --- | --- | --- | --- | --- | --- | --- |
| Hnrnpk | Hnrnpk | 15 | 2 | 4.5 | 3 | 0 | 15.378 | 0 | 1 | 2 | 269100 |
|  | CON__P00761 | 1 | 8 | 49.8 | 120 | 0 | 290.24 | 45 | 53 | 22 | 2123900000 |
|  | CON__P02538 | 4 | 2 | 49.1 | 35 | 0 | 166.58 | 15 | 19 | 1 | 5907900 |
|  | CON__P02769 | 1 | 7 | 16.8 | 16 | 0 | 66.016 | 3 | 11 | 2 | 1224100 |
|  | CON__P04258 | 1 | 1 | 0.8 | 1 | 0 | 8.0135 | 0 | 1 | 0 | 37894 |
|  | CON__P08779 | 5 | 9 | 42.9 | 12 | 0 | 111.01 | 3 | 9 | 0 | 2686200 |
|  | CON__P13717 | 1 | 14 | 54.5 | 142 | 0 | 323.31 | 50 | 58 | 34 | 166780000 |
|  | CON__P48668 | 1 | 0 | 44.7 | 1 | 1 | -2 | 0 | 1 | 0 | 266950 |
|  | CON__Q86YZ3 | 1 | 15 | 14.2 | 29 | 0 | 200.06 | 12 | 16 | 1 | 2668600 |
| Dnah6 | Dnah6 | 2 | 1 | 0.9 | 1 | 0.007752 | 6.3281 | 0 | 1 | 0 | 12198 |
| Col1a1 | Col1a1 | 2 | 2 | 1.5 | 4 | 0 | 20.343 | 1 | 2 | 1 | 630390 |
| Col1a2 | Col1a2 | 2 | 3 | 1.9 | 4 | 0 | 20.057 | 0 | 4 | 0 | 172260 |
| Eif4a2;Eif4a1 | Eif4a2 | 6 | 2 | 5 | 2 | 0 | 12.15 | 0 | 1 | 1 | 163290 |
| Rps13 | Rps13 | 3 | 2 | 13.6 | 2 | 0 | 12.166 | 0 | 2 | 0 | 172170 |

**Supplementary Table S3** Sequences of RNA oligoribonucleotide.

| *Gene* | Strand | Sequence (5’-3’) |
| --- | --- | --- |
| *si1-hnRNPK* | Sense | AAUAUUAAGGCUCUCCGUACATT |
|  | Antisense | UGUACGGCGCGCCUUAAUAUUTT |
| *si2-hnRNPK* | Sense | AAUAUUAAGGCUCUCCGUACATT |
|  | Antisense | UGUACGGAGAGCCUUAAUAUUTT |
| *si3-hnRNPK* | Sense | CAGUGCUGAUAUUGAGACGAUTT |
|  | Antisense | AUCGUCUCAAUAUCAGCACUGTT |
| *si-nc* | Sense | UUCUCCGAACGUGUCACGUTT |
|  | Antisense | ACGUGACACGUUCGGAGAATT |

**Supplementary Table S4** Sequences of ChIRP biotin probes.

| Probe number | Probe sequence 5’---3’  (Antisense of *LITTIP*) | Probe locations (start) |
| --- | --- | --- |
| *LITTIP* Probe 1 | CTAAAATAGCCAACCCTAGG | 42 |
| *LITTIP* Probe 2 | CTCTAAACCTGCAAACCAGC | 131 |
| *LITTIP* Probe 3 | AGATGTAGAACTCTCAGCTC | 212 |
| *LITTIP* Probe 4 | TTGGAGTAGATGGGTCACTG | 303 |
| *LITTIP* Probe 5 | TCCCCAAAAGAATGCCAATT | 388 |
| *LITTIP* Probe 6 | CTTGACTCCAAAGGGGAGAA | 475 |
| *LITTIP* Probe 7 | CACTCTCATAGAAGACCTCT | 559 |
| *LITTIP* Probe 8 | TGGCTGATGTCCAGATTTAG | 639 |
| *LITTIP* Probe 9 | ACTCCTAGCTAGACGTTTAA | 724 |
| *LITTIP* Probe 10 | GAGACAGAGTCTCATGTAGC | 822 |
| *LITTIP* Probe 11 | TACTCAATCTTATTTGGGGT | 902 |
| *LITTIP* Probe 12 | TGGTCTTCAACTGACCACAA | 995 |
| *LITTIP* Probe 13 | ACCAGTCAAAGACTCATGGT | 1011 |
| *LITTIP* Probe 14 | GATTCTTTTCCATGGCTTTG | 1165 |
| *LITTIP* Probe 15 | ACTAATGGCACAGCACACAG | 1247 |
| *LITTIP* Probe 16 | GGGTTTTTTCCTTTTGATGA | 1335 |
| *LITTIP* Probe 17 | GGAGCAGGAGAAGAGGTGAA | 1431 |
| *LITTIP* Probe 18 | CAAGAGCCTACTGCCTATTG | 1518 |
| *LITTIP* Probe 19 | GAGAACTTGGTAACCCAGAG | 1604 |
| *LITTIP* Probe 20 | GTTATAGTTCACATGGCTGG | 1687 |

**Supplementary Table S5**The results of the ChIRP-MS assay.

| **Gene.names** | **Number of proteins** | **Unique peptides** | **Sequence**  **coverage** | **MS.MS.count** | **Q.value** | **Score** | **MS.MS.count.ctrl** | **MS.MS.count.Lnc** | **MS.MS.count.U1** | **iBAQ** | **Symbol** | **Rank** |
| --- | --- | --- | --- | --- | --- | --- | --- | --- | --- | --- | --- | --- |
| Tardbp | 12 | 2 | 8.2 | 2 | 0 | 11.717 | 0 | 0 | 2 | 83363 | Tardbp | 114 |
| Fubp3;Fubp1;Khsrp | 13 | 1 | 4.2 | 1 | 0.007813 | 6.3419 | 1 | 0 | 0 | 0 | Fubp3 | 131.5 |
| Gm8797;Uba52… | 10 | 1 | 20.8 | 3 | 0 | 17.86 | 1 | 1 | 1 | 4515800 | Gm8797 | 22 |
| Vim | 9 | 15 | 38.9 | 45 | 0 | 155.71 | 19 | 9 | 17 | 8710400 | Vim | 15 |
| Srrt | 6 | 1 | 3.7 | 1 | 0 | 7.3913 | 0 | 0 | 1 | 87333 | Srrt | 112 |
| S100a4 | 2 | 1 | 10.3 | 1 | 0 | 6.5955 | 0 | 0 | 1 | 834600 | S100a4 | 50 |
| Serbp1 | 11 | 3 | 27.2 | 5 | 0 | 22.646 | 3 | 0 | 2 | 961000 | Serbp1 | 46 |
| Hist1h2aa;Hist1h2ah… | 24 | 3 | 27.8 | 6 | 0 | 17.085 | 3 | 0 | 3 | 9733100 | Hist1h2aa | 14 |
| Spatc1 | 3 | 1 | 1.9 | 1 | 0 | 6.679 | 0 | 0 | 1 | 309010 | Spatc1 | 82 |
| Tgm1 | 5 | 2 | 3.2 | 2 | 0 | 10.999 | 1 | 0 | 1 | 57805 | Tgm1 | 116 |
| Serpinh1 | 2 | 1 | 7.4 | 1 | 0 | 7.3729 | 1 | 0 | 0 | 225180 | Serpinh1 | 94 |
| Snrnp70 | 4 | 1 | 7.8 | 1 | 0 | 11.662 | 0 | 0 | 1 | 535220 | Snrnp70 | 67 |
| Rpl18 | 7 | 3 | 26.5 | 7 | 0 | 43.819 | 3 | 2 | 2 | 6510900 | Rpl18 | 20 |
| Ldha;Ldhb;Ldhc | 12 | 2 | 7 | 3 | 0 | 13.578 | 1 | 0 | 2 | 586560 | Ldha | 63 |
| Rpsa | 2 | 1 | 12.8 | 2 | 0 | 10.053 | 1 | 0 | 1 | 619310 | Rpsa | 60 |
| Lgals1 | 2 | 2 | 80 | 2 | 0 | 25.531 | 1 | 0 | 1 | 8365900 | Lgals1 | 16 |
| Snrpc | 3 | 1 | 5.7 | 1 | 0 | 9.2697 | 0 | 0 | 1 | 586170 | Snrpc | 64 |
| Matr3 | 7 | 1 | 27.3 | 1 | 0 | 6.4818 | 0 | 0 | 1 | 683780 | Matr3 | 56 |
| Hnrnpa3 | 4 | 3 | 12.9 | 2 | 0 | 17.561 | 1 | 0 | 1 | 198660 | Hnrnpa3 | 96 |
| Rrbp1 | 2 | 3 | 2 | 4 | 0 | 20.493 | 1 | 0 | 3 | 43242 | Rrbp1 | 118 |
| Hnrnpk | 15 | 2 | 4.5 | 3 | 0 | 15.378 | 0 | 1 | 2 | 269100 | Hnrnpk | 89 |
| Flna | 6 | 4 | 1.8 | 5 | 0 | 22.958 | 1 | 0 | 4 | 70839 | Flna | 115 |
| Hnrnpm | 5 | 9 | 14.1 | 6 | 0 | 64.076 | 0 | 0 | 6 | 613900 | Hnrnpm | 62 |
| Rpl15 | 2 | 1 | 8.4 | 2 | 0.007874 | 6.356 | 1 | 0 | 1 | 997810 | Rpl15 | 44 |
|  | 1 | 0 | 16 | 2 | 0 | 7.1848 | 1 | 1 | 0 | 176830 | CON__ENSEMBL:ENSBTAP00000038253 | 99 |
|  | 1 | 8 | 49.8 | 120 | 0 | 290.24 | 45 | 53 | 22 | 2123900000 | CON__P00761 | 1 |
|  | 13 | 5 | 51.1 | 82 | 0 | 323.31 | 34 | 34 | 14 | 27688000 | CON__P02533 | 11 |
|  | 4 | 2 | 49.1 | 35 | 0 | 166.58 | 15 | 19 | 1 | 5907900 | CON__P02538 | 21 |
|  | 1 | 3 | 9.4 | 5 | 0 | 17.761 | 1 | 1 | 3 | 538070 | CON__P02768-1 | 65 |
|  | 1 | 7 | 16.8 | 16 | 0 | 66.016 | 3 | 11 | 2 | 1224100 | CON__P02769 | 39 |
|  | 1 | 1 | 0.8 | 1 | 0 | 8.0135 | 0 | 1 | 0 | 37894 | CON__P04258 | 119 |
|  | 1 | 25 | 57.6 | 414 | 0 | 323.31 | 190 | 128 | 96 | 497390000 | CON__P04264 | 3 |
|  | 5 | 9 | 42.9 | 12 | 0 | 111.01 | 3 | 9 | 0 | 2686200 | CON__P08779 | 25 |
|  | 38 | 25 | 54.6 | 353 | 0 | 323.31 | 175 | 87 | 91 | 466840000 | CON__P13645 | 4 |
|  | 18 | 6 | 47.8 | 85 | 0 | 299.9 | 41 | 20 | 24 | 28978000 | CON__P13647 | 10 |
|  | 1 | 14 | 54.5 | 142 | 0 | 323.31 | 50 | 58 | 34 | 166780000 | CON__P13717 | 5 |
|  | 1 | 2 | 0.4 | 2 | 0 | 11.649 | 1 | 0 | 1 | 31969 | CON__P20930 | 121 |
|  | 1 | 27 | 53 | 189 | 0 | 323.31 | 96 | 51 | 42 | 156610000 | CON__P35527 | 7 |
|  | 4 | 29 | 68.7 | 247 | 0 | 323.31 | 106 | 67 | 74 | 162350000 | CON__P35908 | 6 |
|  | 1 | 0 | 44.7 | 1 | 1 | -2 | 0 | 1 | 0 | 266950 | CON__P48668 | 90 |
| Krt17 | 6 | 2 | 25.9 | 5 | 0 | 18.472 | 2 | 2 | 1 | 282540 | Krt17 | 84 |
|  | 1 | 7 | 5.6 | 13 | 0 | 61.176 | 5 | 4 | 4 | 1993200 | CON__Q5D862 | 34 |
|  | 5 | 3 | 6.9 | 9 | 0 | 20.584 | 3 | 3 | 3 | 423020 | CON__Q6KB66-1 | 74 |
|  | 3 | 0 | 7.2 | 19 | 0 | 46.103 | 8 | 5 | 6 | 4413200 | CON__Q7RTS7 | 23 |
|  | 1 | 3 | 11.4 | 3 | 0 | 25.428 | 1 | 0 | 2 | 475820 | CON__Q7Z794 | 70 |
|  | 1 | 15 | 14.2 | 29 | 0 | 200.06 | 12 | 16 | 1 | 2668600 | CON__Q86YZ3 | 26 |
|  | 2 | 10 | 27.3 | 12 | 0 | 67.831 | 8 | 2 | 2 | 2751200 | CON__Q8N1N4-2 | 24 |
|  | 1 | 4 | 43.1 | 140 | 0 | 289.02 | 58 | 38 | 44 | 575610000 | CON__Streptavidin | 2 |
| Akr1b3;Akr1b1 | 2 | 2 | 12.5 | 1 | 0 | 12.955 | 0 | 0 | 1 | 0 | Akr1b3 | 131.5 |
| Ywhaz | 11 | 2 | 68.2 | 4 | 0 | 18.366 | 2 | 0 | 2 | 6782600 | Ywhaz | 18 |
| Cav1 | 5 | 1 | 17 | 2 | 0.007692 | 6.2763 | 1 | 1 | 0 | 304320 | Cav1 | 83 |
| Atp5a1 | 3 | 6 | 14.9 | 9 | 0 | 39.975 | 5 | 0 | 4 | 446730 | Atp5a1 | 72 |
| Tpm3;Tpm3-rs7 | 8 | 3 | 12.6 | 5 | 0 | 17.813 | 1 | 1 | 3 | 491440 | Tpm3 | 69 |
| Dnah6 | 2 | 1 | 0.9 | 1 | 0.007752 | 6.3281 | 0 | 1 | 0 | 12198 | Dnah6 | 122 |
| Plec | 17 | 9 | 1.9 | 8 | 0 | 52.049 | 1 | 0 | 7 | 48463 | Plec | 117 |
| Dsp | 2 | 23 | 7.4 | 36 | 0 | 143.79 | 17 | 10 | 9 | 747300 | Dsp | 54 |
| Ahnak | 4 | 4 | 5.8 | 6 | 0 | 26.728 | 2 | 2 | 2 | 84073 | Ahnak | 113 |
| Cald1 | 10 | 2 | 8.5 | 2 | 0 | 11.401 | 0 | 0 | 2 | 521010 | Cald1 | 68 |
| Sprr2k… | 7 | 1 | 13.2 | 7 | 0 | 8.5688 | 3 | 2 | 2 | 1555800 | Sprr2k | 36 |
| Srsf1 | 5 | 2 | 6.7 | 1 | 0 | 11.382 | 0 | 0 | 1 | 245050 | Srsf1 | 92 |
| Tpi1 | 2 | 2 | 15 | 3 | 0 | 11.47 | 2 | 0 | 1 | 309790 | Tpi1 | 81 |
| Phb2 | 2 | 2 | 7 | 2 | 0 | 17.11 | 0 | 0 | 2 | 282070 | Phb2 | 85 |
| Hnrnpa2b1 | 4 | 4 | 13.3 | 3 | 0 | 30.05 | 1 | 0 | 2 | 837390 | Hnrnpa2b1 | 49 |
| Rbm3 | 2 | 2 | 11.8 | 2 | 0 | 19.702 | 0 | 0 | 2 | 982410 | Rbm3 | 45 |
| Anxa2 | 4 | 6 | 18.9 | 8 | 0 | 39.678 | 3 | 2 | 3 | 2192200 | Anxa2 | 31 |
| Hsp90b1 | 2 | 3 | 5 | 6 | 0 | 21.71 | 3 | 0 | 3 | 187420 | Hsp90b1 | 98 |
| P4hb | 2 | 2 | 5.5 | 3 | 0 | 13.047 | 2 | 0 | 1 | 111220 | P4hb | 109 |
| Ncl | 1 | 3 | 4.7 | 5 | 0 | 21.216 | 2 | 0 | 3 | 324460 | Ncl | 80 |
| Pgk1;Pgk2 | 3 | 3 | 5.8 | 5 | 0 | 20.391 | 3 | 0 | 2 | 358110 | Pgk1 | 78 |
| Eef1a1;Eef1a2 | 4 | 4 | 8.7 | 18 | 0 | 28.619 | 7 | 3 | 8 | 2548900 | Eef1a1 | 28 |
| Txn | 1 | 1 | 8.6 | 1 | 0 | 7.4468 | 0 | 0 | 1 | 2560700 | Txn | 27 |
| Col1a1 | 2 | 2 | 1.5 | 4 | 0 | 20.343 | 1 | 2 | 1 | 630390 | Col1a1 | 59 |
| Hsp90ab1 | 7 | 9 | 14.8 | 20 | 0 | 68.627 | 9 | 3 | 8 | 1825900 | Hsp90ab1 | 35 |
| Calr | 1 | 1 | 2.2 | 1 | 0 | 11.483 | 0 | 0 | 1 | 271280 | Calr | 87 |
| Eno1 | 15 | 3 | 7.8 | 6 | 0 | 20.499 | 2 | 1 | 3 | 813680 | Eno1 | 53 |
| Hspa5 | 1 | 9 | 18.6 | 23 | 0 | 81.386 | 11 | 2 | 10 | 2075300 | Hspa5 | 33 |
| Msn | 3 | 3 | 4.9 | 4 | 0 | 17.522 | 1 | 0 | 3 | 153030 | Msn | 105 |
| Pdia3 | 2 | 2 | 4.2 | 1 | 0 | 11.181 | 0 | 0 | 1 | 221050 | Pdia3 | 95 |
| Hspa9 | 1 | 2 | 2.7 | 5 | 0 | 19.873 | 2 | 1 | 2 | 460630 | Hspa9 | 71 |
| Hist1h1e… | 5 | 2 | 16.4 | 3 | 0 | 18.236 | 1 | 1 | 1 | 2311200 | Hist1h1e | 30 |
| Rpl13 | 1 | 4 | 17.5 | 9 | 0 | 57.36 | 4 | 3 | 2 | 2469700 | Rpl13 | 29 |
| Lmna | 8 | 3 | 5.1 | 7 | 0 | 18.672 | 3 | 1 | 3 | 250770 | Lmna | 91 |
| Pkm | 9 | 8 | 15.3 | 14 | 0 | 57.232 | 5 | 1 | 8 | 829980 | Pkm | 51 |
| Atp5b | 1 | 4 | 9.8 | 4 | 0 | 25.255 | 3 | 0 | 1 | 641050 | Atp5b | 58 |
| Eef2 | 1 | 2 | 2.2 | 4 | 0 | 12.663 | 2 | 0 | 2 | 161600 | Eef2 | 104 |
| Actb;Actg1… | 23 | 13 | 41.1 | 32 | 0 | 134.24 | 12 | 7 | 13 | 17184000 | Actb | 13 |
| Rps14 | 3 | 2 | 15.9 | 4 | 0 | 12.999 | 1 | 1 | 2 | 1140900 | Rps14 | 40 |
| Hist1h4a | 1 | 4 | 40.8 | 10 | 0 | 27.399 | 4 | 4 | 2 | 8082700 | Hist1h4a | 17 |
| Hspd1 | 2 | 3 | 8.5 | 7 | 0 | 27.135 | 3 | 2 | 2 | 1056300 | Hspd1 | 42 |
| Tuba1c;Tuba1a… | 10 | 3 | 5.8 | 4 | 0 | 22.779 | 2 | 1 | 1 | 706360 | Tuba1c | 55 |
| Tubb5;Tubb2b… | 5 | 1 | 7.2 | 3 | 0 | 19.917 | 2 | 0 | 1 | 176530 | Tubb5 | 100 |
| Col1a2 | 2 | 3 | 1.9 | 4 | 0 | 20.057 | 0 | 4 | 0 | 172260 | Col1a2 | 101 |
| Vcp | 1 | 2 | 2.9 | 2 | 0 | 11.185 | 1 | 0 | 1 | 148030 | Vcp | 107 |
| Jup | 3 | 15 | 24 | 20 | 0 | 106.16 | 10 | 6 | 4 | 2156600 | Jup | 32 |
| 1700009N14Rik;Ran | 2 | 1 | 5.1 | 1 | 0 | 7.2027 | 0 | 0 | 1 | 435380 | 1700009N14Rik | 73 |
| Pnn | 2 | 1 | 1.2 | 1 | 0.007519 | 6.1574 | 0 | 0 | 1 | 0 | Pnn | 131.5 |
| Ddx17 | 2 | 1 | 4 | 1 | 0 | 6.4552 | 0 | 0 | 1 | 0 | Ddx17 | 131.5 |
| Krt76 | 1 | 1 | 10.4 | 9 | 0 | 6.7313 | 5 | 0 | 4 | 90504000 | Krt76 | 9 |
| Hspa8;Hspa2 | 7 | 6 | 13.4 | 10 | 0 | 42.385 | 4 | 2 | 4 | 1076500 | Hspa8 | 41 |
| Srsf4 | 2 | 1 | 2.6 | 1 | 0 | 7.1882 | 0 | 0 | 1 | 0 | Srsf4 | 131.5 |
| Hnrnpa1 | 2 | 1 | 4.8 | 2 | 0 | 27.667 | 1 | 0 | 1 | 238020 | Hnrnpa1 | 93 |
| Psmb6 | 1 | 2 | 8.8 | 4 | 0 | 12.073 | 2 | 0 | 2 | 269700 | Psmb6 | 88 |
| Naca | 2 | 1 | 7 | 1 | 0 | 7.644 | 0 | 0 | 1 | 0 | Naca | 131.5 |
| Fscn1 | 3 | 2 | 3.7 | 3 | 0 | 11.474 | 2 | 0 | 1 | 192210 | Fscn1 | 97 |
| Srsf2 | 1 | 2 | 6.8 | 2 | 0 | 12.117 | 0 | 0 | 2 | 1011200 | Srsf2 | 43 |
| Ddx3x;Ddx3y… | 3 | 2 | 3.8 | 2 | 0 | 13.518 | 0 | 0 | 2 | 92569 | Ddx3x | 111 |
| Ccdc18 | 1 | 1 | 2.7 | 1 | 0 | 6.7713 | 0 | 0 | 1 | 279910 | Ccdc18 | 86 |
| Tpm4 | 2 | 2 | 7.3 | 2 | 0 | 11.556 | 0 | 0 | 2 | 0 | Tpm4 | 131.5 |
| Rps9 | 1 | 2 | 12.9 | 2 | 0 | 11.434 | 1 | 0 | 1 | 537950 | Rps9 | 66 |
| Hist1h2bc;Hist2h2bb… | 4 | 1 | 19.8 | 3 | 0 | 6.9492 | 1 | 1 | 1 | 844600 | Hist1h2bc | 48 |
| Eif4a2;Eif4a1 | 6 | 2 | 5 | 2 | 0 | 12.15 | 0 | 1 | 1 | 163290 | Eif4a2 | 103 |
| Hnrnph1;Hnrnph2 | 3 | 1 | 3.6 | 2 | 0 | 10.187 | 1 | 0 | 1 | 648610 | Hnrnph1 | 57 |
| Hist1h2br;Hist1h2bp… | 10 | 1 | 18.7 | 7 | 0 | 19.416 | 3 | 2 | 2 | 6603300 | Hist1h2br | 19 |
| Myh9 | 1 | 2 | 1.1 | 2 | 0 | 12.518 | 0 | 0 | 2 | 34304 | Myh9 | 120 |
| Sfpq;Nono | 3 | 2 | 3.3 | 2 | 0 | 11.146 | 0 | 0 | 2 | 372550 | Sfpq | 77 |
| Rps13 | 3 | 2 | 13.6 | 2 | 0 | 12.166 | 0 | 2 | 0 | 172170 | Rps13 | 102 |
| Pdia6 | 1 | 1 | 3.4 | 1 | 0 | 7.526 | 0 | 0 | 1 | 0 | Pdia6 | 131.5 |
| 2210010C04Rik | 1 | 1 | 4.9 | 6 | 0 | 8.3942 | 2 | 2 | 2 | 25489000 | 2210010C04Rik | 12 |
| Aldoart1;Aldoa… | 10 | 2 | 6.9 | 2 | 0 | 11.714 | 2 | 0 | 0 | 94772 | Aldoart1 | 110 |
| Rps19 | 6 | 3 | 17.9 | 4 | 0 | 21.598 | 3 | 0 | 1 | 417750 | Rps19 | 75 |
| S100a14 | 1 | 1 | 14.4 | 2 | 0.007407 | 6.1073 | 1 | 0 | 1 | 0 | S100a14 | 131.5 |
| Atp5d | 1 | 1 | 8.3 | 1 | 0 | 7.3467 | 1 | 0 | 0 | 343180 | Atp5d | 79 |
| Npm1 | 4 | 1 | 3.5 | 1 | 0 | 7.0697 | 1 | 0 | 0 | 1274100 | Npm1 | 38 |
| Olfr704;Olfr694… | 7 | 1 | 2.2 | 4 | 0.007634 | 6.1995 | 2 | 1 | 1 | 105410000 | Olfr704 | 8 |
| Cpn10-rs1;Hspe1 | 2 | 1 | 13.7 | 1 | 0 | 7.2174 | 1 | 0 | 0 | 821320 | Cpn10-rs1 | 52 |
| Plp2 | 1 | 1 | 7.9 | 3 | 0.007463 | 6.121 | 1 | 0 | 2 | 956840 | Plp2 | 47 |
| Tagln2 | 2 | 2 | 10.6 | 2 | 0 | 11.559 | 0 | 0 | 2 | 143600 | Tagln2 | 108 |
| Hnrnpc | 8 | 3 | 10.6 | 2 | 0 | 22.687 | 0 | 0 | 2 | 149260 | Hnrnpc | 106 |
| Ddx5;Ddx17 | 7 | 1 | 5.2 | 3 | 0 | 14.222 | 1 | 0 | 2 | 379450 | Ddx5 | 76 |
| Gm3839… | 9 | 4 | 14.4 | 12 | 0 | 28.453 | 4 | 2 | 6 | 1483500 | Gm3839 | 37 |

**Supplementary Table S6** Sequences of primers used in qRT-PCR.

| *Gene* | Forward primer sequence (5’-3’) | Reverse primer sequence (5’-3’) |
| --- | --- | --- |
| *Gapdh* | AGGTCGGTGTGAACGGATTTG | TGTAGACCATGTAGTTGAGGTCA |
| *Runx2* | CCTTCAAGGTTGTAGCCCTC | GGAGTAGTTCTCATCATTCCCG |
| *Alp* | TCATTCCCACGTTTTCACATTC | GTTGTTGTGAGCGTAATCTACC |
| *ColA1* | TGAACGTGGTGTACAAGGTC | CCATCTTTACCAGGAGAACCAT |
| *Hacd1* | CCTGGCTCACCTTCTACAATAT | CAATTCCGATCAGACAATGGAC |
| *Ctnnb1* | TGCCGTTCGCCTTCATTATGGAC | TGGGCAAAGGGCAAGGTTTCG |
| *Axin2* | GTCCTGGGGGAACAGATTATTA | CATTCCGTTTTGGCAAGGTAC |
| *APC* | AAGTACTTAAGCAGCTACAGGG | GGGACATTTTTGAGCGTAGTTT |
| *Sp7* | TCGTCTGACTGCCTGCCTAGTG | CTGCGTGGATGCCTGCCTTG |
| *Lgr6* | CACCTCTGGCTGGATGACAATGC | TAGTCAGGGATGTGGCGGATATGG |
| *HnRNPK* | GAAGTGACTTTGATTGCGAGTT | GCTTGATTGTTGTCTGAGTGTT |
| *AK032137* | CTGGCAATAGGCAGTAGGCTCTTG | GAGGTGGAGGTGGTAGGTCTTAGG |
| *U6* | GAAGATTTAGCATGGCCCCTGC | CAGTGCAGGGTCCGAGGT |
